# The Molecular Mechanism of Interaction Between SEPALLATA3 and APETALA1 in *Arabidopsis thaliana*

**DOI:** 10.1002/pld3.70052

**Published:** 2025-03-30

**Authors:** Xiao‐Min Tan, Ya‐Ru Li, Man‐Ru Song, Ling‐Na Yuan, Zi‐Xin Zhao, Ye Liu, Qi Meng, Xuan Huang, Ye‐Ye Ma, Zi‐Qin Xu

**Affiliations:** ^1^ Key Laboratory of Resource Biology and Biotechnology in Western China (Ministry of Education), Shaanxi Provincial Key Laboratory of Biotechnology College of Life Sciences, Northwest University Xi'an Shaanxi People's Republic of China

**Keywords:** *AtAP1* gene, *AtSEP3* gene, K1 subdomain, petal, protein interaction, site‐specific mutation

## Abstract

Flower formation has been a primary focus in botanical research, leading to the identification of multiple factors regulating flowering over the past 30 years. The MADS transcription factors SEPALLATA3 (SEP3) and APETALA1 (AP1) are essential for floral meristem development and organ identity. In Arabidopsis, SEP3 functions as a central integrator, combining MADS proteins into a tetrameric complex, with its interaction with AP1 playing a key role in sepal and petal formation. This research explores *AtSEP3* and *AtAP1*, with particular emphasis on the Leu residue in the K1 subfunctional domain of *AtSEP3*, which is necessary for their interaction. A predicted structural model of AP1 was used, followed by protein docking with SEP3, which indicated that Leu residues at positions 115 and 116 are critical binding sites. Mutations at these position were examined through yeast two‐hybrid assays and other techniques, identifying Leu 116 as a significant site. Subsequent purification and EMSA analysis revealed that mutations in the leucine zipper of SEP3 decreased its DNA binding ability. Observations of transgenic plants showed that disruption of *AtSEP3* and *AtAP1* interaction resulted in extended vegetative growth, increased size and number of rosette leaves, and modifications in floral structures. This study offers new insights into the interaction mechanism between AP1 and SEP3 during flowering.

## 1 Introduction

The transition from vegetative growth to reproductive growth and the quality of development in higher plants are significantly influenced by flower development. Flowering serves as a critical juncture in plant development, playing an essential role in determining the overall growth trajectory. Both internal and external environmental factors contribute to the regulation of flowering, ensuring it occurs at the appropriate time (Xiao et al. 2022).

The MADS‐box gene family is an important internal factor that regulates the flowering process (Abdullah‐Zawawi et al. 2021). These genes are involved in development process of floral organs and the regulation of flowering as well as impacting the growth of fruits, roots, stems, and leaves.

MIKC‐type MADS‐box proteins in plants are characterized by conserved domains, which mainly consist of four primary regions (Du et al. 2024). The MADS‐box (M) domain is the most conserved, whereas the Intervening (I) domain, which aids in dimer formation of dimers during DNA binding, is less conserved. The K‐box (K) domain is crucial for protein interactions, helping to form coiled‐coil structures that facilitate dimerization and interactions between proteins. It is also involved in the assembly of higher order protein complexes. The C‐terminal (C) domain, located downstream of the K domain, is the least conserved and is important for the formation of multitranscription factor complexes and the activation of transcriptional functions (Fu et al. 2024).

The flowering process marks a critical phase in the shift from vegetative to reproductive growth in plants, with AP1 (APETALA1) from the Class A family acting as a key determinant gene for bud differentiation (Zhang et al. 2021), typically expressed downstream of LFY (Hu et al. 2023). AP1 is essential for inducing flowering and plays a significant role in the transition of the inflorescence meristem (Chen and Du 2022), also regulating the formation of the floral meristem. In *ap1* mutants, sepals are converted into leaf‐like structures, and petals fail to develop.

Among the MADS‐box family genes, the *SEPALLATA* genes appear later in the process. Evolutionary studies suggest that the E‐class family plays a significant role in the evolution of flowering plants from their nonflowering ancestors (Wang et al. 2022). SEPALLATA proteins serve as a central hub of in MADS complexes during the flowering process, particularly participating in the MADS transcription factors responsible for flowering and floral organ development (Dreni and Ferrándiz 2022). Of the four *SEP* genes, *SEP3* has been the most extensively researched (Käppel et al. 2018; Osnato et al. 2021). *SEP3* contributes to the formation of complexes that determine floral organ identity and is also involved in controlling the transition to flowering, flowering time, and ovule development (Smaczniak et al. 2012). Flowers from *sep3‐1* and *sep3‐2* single mutant plants exhibit partial transformation of petals into sepals, and axillary flowers seldomly develop at the base of the first‐whorl sepals (Pelaz et al. 2001).

Prior to the initiation of the flowering transition, the FLOWERING LOCUS C (FLC) ‐ SHORT VEGETATIVE PHASE (SVP complex suppresses the flowering integrators SUPPRESSOR OF OVEREXPRESSION OF CO1 (SOC1 and FLOWERING LOCUS T (FT) at the stem tip (Lee et al. 2021; Posé et al. 2012; Shen et al. 2021), whereas *AtAP1* and LEAFY (LFY are inhibited by TERMINAL FLOWER 1 (TFL1), thereby obstructing the development of inflorescence meristematic tissue (Mateos et al. 2015). When FLC expression is downregulated due to external factors such as cold, the interaction partner of SVP shifts from FLC to FUL, leading to the activation of SOC1, whose protein product subsequently interacts with FUL (Liu et al. 2022; Serrano‐Mislata et al. 2017). At this stage, the floral transition is further regulated by the AGL24‐SOC1 dimer, which contributes to inflorescence meristem organization through a positive feedback loop (Song et al. 2023). Both AGAMOUS‐LIKE 24 (AGL24)‐SOC1 and FUL‐SOC1 dimers are proposed to induce *LFY* expression, which then stimulates AP1 expression, ultimately initiating the organization of inflorescence meristem (Mateos et al. 2015). In newly formed floral meristematic tissues, the dimerization of AP1 with SOC1, AGL24, and SVP suppresses *TFL1* (Huang et al. 2024).

To prevent premature floral organ development, *SEP3* is inhibited by the flowering time regulators AGL24, SVP, and SOC1, along with the auxin repressor complex comprising AP1‐AGL24, SEUSS (SEU)‐LEUNIG (LUG), and AP1‐SVP (Yang et al. 2019; Lee et al. 2022). When SEP3 expression accumulates during the third stage of flowering, driven by AP1 and LFY activation, it begins to suppress flowering time genes in collaboration with AP1 (Gregis et al. 2009). This suppression likely involves negative feedback regulation mediated by the SEP3‐SOC1, SEP3‐SVP, and SEP3‐AGL24 complexes. The SEP3‐AP1 dimer, in conjunction with LFY, activates floral organ identity genes *AG*, *AP3*, and *PI* (Kaufmann et al. 2009). The AP1‐SEP3 complex, identified in *Arabidopsis* with the elusive A function in sepal and petal formation, is associated with the transition from regulating floral meristem identity to controlling floral organ identity, contributing to the development of sepals and petals (Li et al. 2023).

Despite significant progress, the biochemical and biophysical mechanisms that enable SEP3 to form DNA‐binding dimers and tetramers with diverse partners, whereas other MIKC‐type MADS domain transcription factors are unable to form tetramer‐like complexes independently, remain unclear. For example, the floral homeotic proteins AP3 and PI, which regulate petal and stamen development in *Arabidopsis*, can only form obligate heterodimers and require SEP proteins to facilitate tetramer formation (Yang et al. 2003; Käppel et al. 2018). Critical questions persist regarding the floral development tetramer model. Furthermore, given the pivotal roles of *AtSEP3* and *AtAP1* in the flowering transition and floral organ development in *Arabidopsis*, further investigation into the interaction between these proteins and their functional impact on these processes is imperative.

## 2 Materials and Methods

### 2.1 Plant Material

Wild‐type *Arabidopsis thaliana* (Col‐0) was cultivated under a 16‐h light/8‐h dark cycle in a climate‐controlled environment at 25°C. Tobacco plants were grown in an artificial climate chamber with a 14‐h light/10‐h dark cycle at an ambient temperature of 25°C.

### 2.2 Cloning of the Coding Sequences of *AtSEP3* and *AtAP1*

RNA was isolated using the TRIzol reagent and reversely transcribed into cDNA with the Evo M‐MLV Reverse Transcription Premix Kit (AG11728). Coding sequences of *AtSEP3* (AT1G24260) and *AtAP1* (AT1G69120) were amplified using high‐fidelity PrimeSTAR Max DNA Polymerase. To address the absence of the K3 coding region in the *AtSEP3.3* splicing variant, overlapping PCR was employed. Based on sequence comparison with *AtSEP3.2*, two primers containing overlapping fragments were designed to bridge gap. PCR was conducted using 5′‐ and 3′‐terminal primers, with the resulting fragments mixed in equal concentrations and subjected to a thermal cycling protocol.

**FIGURE 1:**
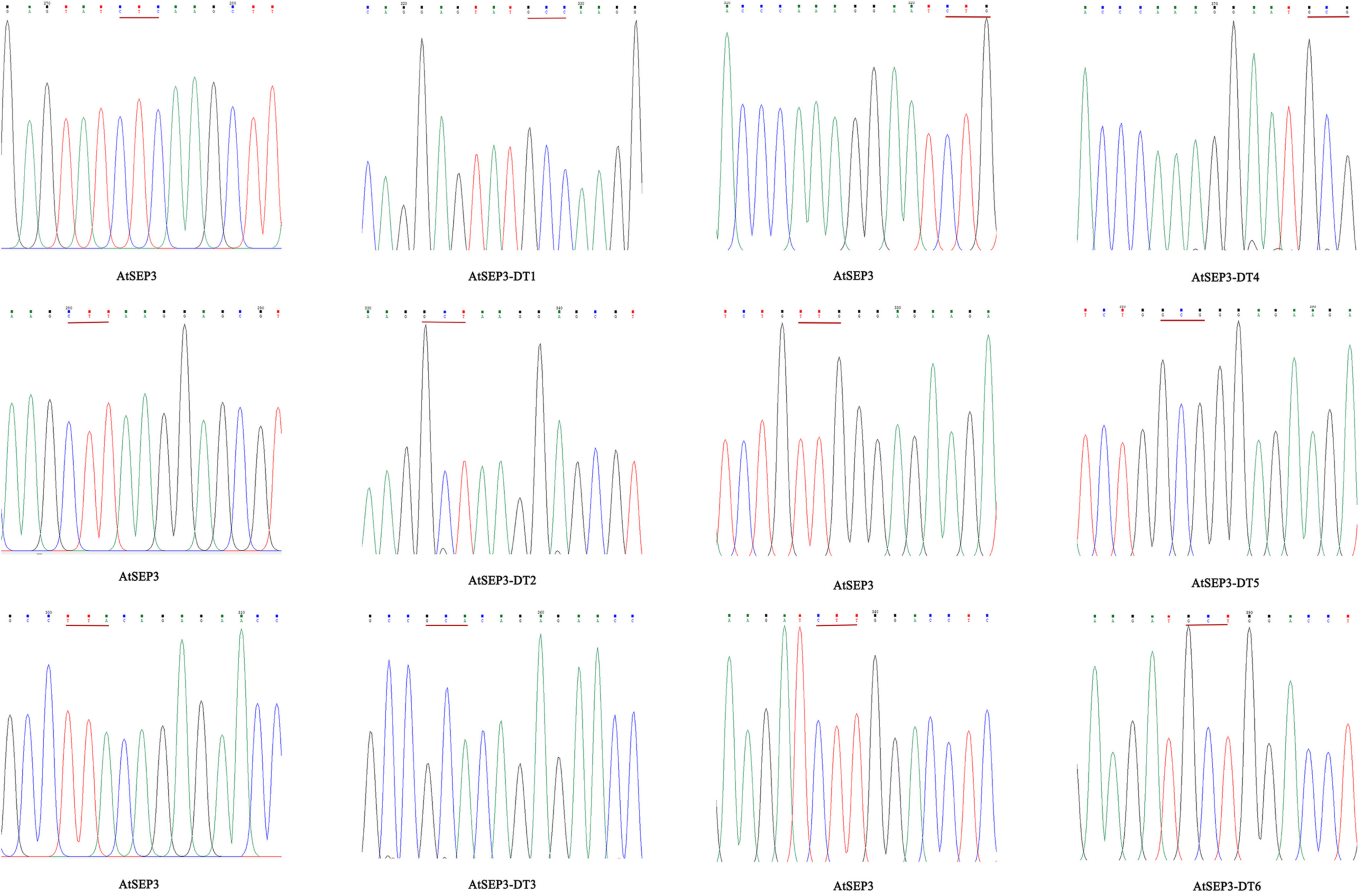
Sequence comparison plot of single point mutations.

**FIGURE 2:**
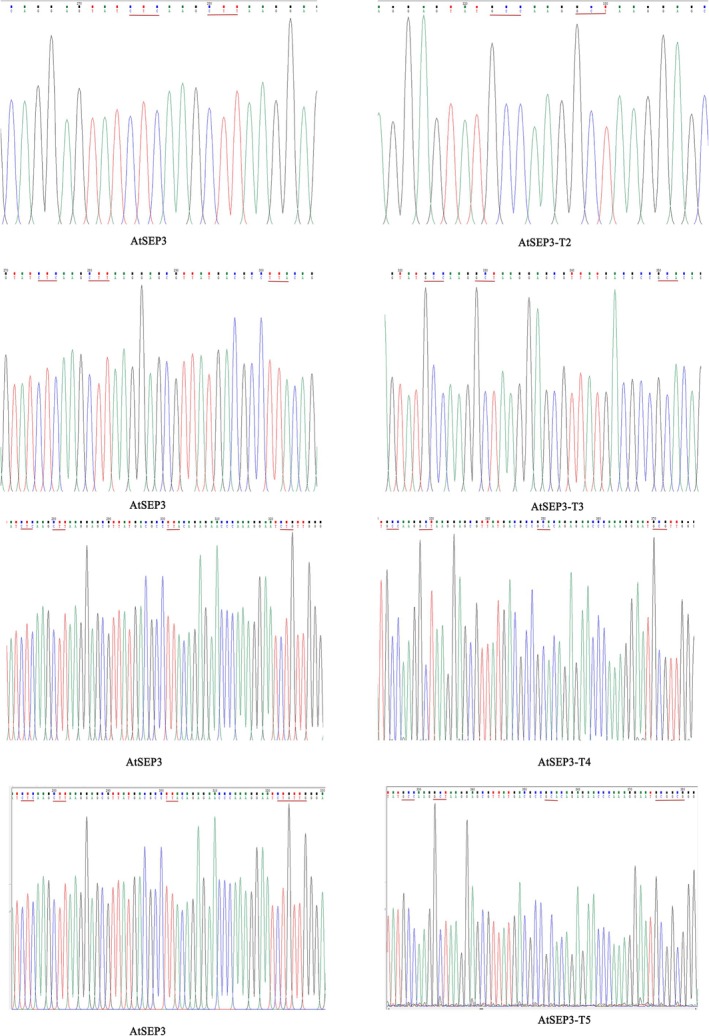
Sequence comparison plot of multiple point mutations.

For the construction of pGADT7‐*AtSEP3*, primers incorporating *NdeI* and *EcoRI* restriction sites were designed, whereas primers for pGBKT7‐*AtAP1* included *NdeI* and *BamHI* sites. In total, 10 pGADT7‐based *AtSEP3* yeast vectors were generated, including a vector containing the intact open reading frame (ORF) from the first splice copy of *AtSEP3* (1–756 bp), along with vectors carrying specific mutations. These mutations included single‐site mutations (Leu to Ala at position 99), double‐site mutations (Leu to Ala at positions 99 and 101), triple‐site mutations (Leu to Ala at positions 99, 101, and 108), four‐site mutations (Leu to Ala at positions 99, 101, 108, and 115), and five‐site mutations (Leu to Ala at positions 99, 101, 108, 115, and 116). The multisite mutations were labeled *AtSEP3*‐T1/2/3/4/5 based on the number of mutated leucine residues, whereas single‐site mutations were designated *AtSEP3*‐DT1/2/3/4/5/6, as shown in Figures 1 and 2. All recombinant plasmids incorporated *NdeI* and *EcoRI* as upstream and downstream restriction sites. Similarly, *BamHI* and *SalI* sites were introduced into primers for constructing pSPYCE‐35S/PUC‐*AtSEP3*, pSPYCE‐35S/PUC‐*AtSEP3‐T5*, and pSPYNE‐35S/PUC‐*AtAP1* (Table 1).

**TABLE 1:** Primers used for construction of protein–protein interaction related vectors.

Primer name	Sequences (5′ → 3′)
pGADT7‐*AtSEP3*‐F	GCGCATATGGGAAGAGGGAGAGTAGAATTG
pGADT7‐*AtSEP3*‐R	CGCGAATTCAATAGAGTTGGTGTCATAAGG
pGBKT7‐*AtAP1*‐F	GCGCATATGGGAAGGGGTAGGGTTCAATT
pGBKT7‐*AtAP1*‐R	CGCGGATCCTGCGGCGAAGCAGCCAAGGT
pGADT7‐*SEP3‐T1*‐F	CAGCAGGAGTAT*GCC*AAGCTTAAGGAGCGT
pGADT7‐*SEP3‐T1*‐R	ACGCTCCTTAAGCTTGGCATACTCCTGCTG
pGADT7‐*SEP3‐T2*‐F	AGTAT*GCC*AAG*GCT*AAGGAGCGTTATGACG
pGADT7‐*SEP3‐T2*‐R	CGTCATAACGCTCCTTAGCCTTGGCATACT
pGADT7‐*SEP3‐T3*‐F	AGGAGCGTTATGACGCC*GCA*CAGAGAACCC
pGADT7‐*SEP3‐T3*‐R	GGGTTCTCTGTGCGGCGTCATAACGCTCCT
pGADT7‐*SEP3‐T4*‐F	CCCAAAGGAAT*GCG*TTGGGAGAAGATCTTG
pGADT7‐*SEP3‐T4*‐R	CAAGATCTTCTCCCAACGCATTCCTTTGGG
pGADT7‐*SEP3‐T5*‐F	GAGAACCCAAAGGAATGCG*GCG*GGAGAAGATCTTGGACCTC
pGADT7‐*SEP3‐T5*‐R	GAGGTCCAAGATCTTCTCCCGCCGCATTCCTTTGGGTTCTC
pGADT7‐*SEP3‐DT2*‐F	AGTATCTCAAG*GCT*AAGGAGCGTTATGACG
pGADT7‐*SEP3‐DT2*‐R	CGTCATAACGCTCCTTAGCCTTGAGATACT
pGADT7‐*SEP3‐DT3*‐F	AGGAGCGTTATGACGCC*GCA*CAGAGAACCC
pGADT7‐*SEP3‐DT3*‐R	GGGTTCTCTGTGCGGCGTCATAACGCTCCT
pGADT7‐*SEP3‐DT4*‐F	CAGAGAACCCAAAGGAAT*GCG*TTGGGAGAAGATCTTGG
pGADT7‐*SEP3‐DT4*‐R	CCAAGATCTTCTCCCAACGCATTCCTTTGGGTTCTCTG
pGADT7‐*SEP3‐DT5*‐F	CCCAAAGGAATCTG*GCG*GGAGAAGATCTTG
pGADT7‐*SEP3‐DT5*‐R	CAAGATCTTCTCCCGCCAGATTCCTTTGGG
pGADT7‐*SEP3‐DT6*‐F	TCTGTTGGGAGAAGAT*GCT*GGACCTCTAAGTA
pGADT7‐*SEP3‐DT6*‐R	GTACTTAGAGGTCCAGCATCTTCTCCCAACAGA
pSPYCE‐35S/PUC‐*AtSEP3*‐F	GCGACTAGTGGAAGAGGGAGAGTAGAATTGAAG
pSPYCE‐35S/PUC‐*AtSEP3*‐R	CGCGTCGACAATAGAGTTGGTGTCATAAGGTAAC
pSPYCE‐35S/PUC‐*AtSEP3‐T5*‐F	GCGACTAGTGGAAGAGGGAGAGTAGAATTGAAG
pSPYCE‐35S/PUC‐*AtSEP3‐T5*‐R	CGCGTCGACAATAGAGTTGGTGTCATAAGGTAAC
pSPYNE‐35S/PUC‐*AtAP1*‐F	GCGACTAGTATGGGAAGGGGTAGGGTTC
pSPYNE‐35S/PUC‐*AtAP1*‐R	CGCGTCGACTGCGGCGAAGCAGCCAAGG
pGADT7‐*SEP3‐K1*‐ad‐F	GCGCATATGGAACTTAGTAGCCAGCAGGAG
pGADT7‐*SEP3‐K1*‐ad‐R	CGCGAATTCCAACAGATTCCTTTGGGTTCTC
pGADT7‐*SEP3‐K2*‐ad‐F	GCGCATATGAGTACAAAGGAGCTTGAGTC
pGADT7‐*SEP3‐K2*‐ad‐R	CGCGAATTCCTGTGTCCTGAGAGCTCTGA
*SEP3.3*‐prime‐F	CAACGATCTTCAGAGTAAGTTAGCTGATGGGTATC
*SEP3.3*‐prime‐R	GATACCCATCAGCTAACTTACTCTGAAGATCGTTG

*Note:* The mutant amino acid sites are marked in italics.

### 2.3 Site‐Directed Mutagenesis and β‐Galactosidase Activity Detection

Site‐directed mutagenesis was conducted performed on the complete SEP3 open reading frame (ORF) using pGADT7 vectors to generate a series of mutants, such as SEP3‐L99A and others with multiple site ‐specific alterations. The individual mutations were introduced using primers incorporating *NdeI* and *EcoRI* restriction sites, facilitating the construction of the desired mutant variants.

PCR was performed using the pGADT7‐*AtSEP* template, followed by demethylation with *DpnI* to eliminate the methylated plasmid templates. The resulting product was transformed into *Escherichia coli* DH5α competent cells, which were transformed and cultured overnight. Monoclonal colonies were subsequently verified through colony PCR and sequencing to confirm successful mutagenesis.

Yeast colonies were cultured on deficient media and expanded in liquid culture until an OD_600_ of 0.6–0.8was achieved. For the assay preparation, yeast samples were centrifuged and resuspended in Z‐buffer (prepared by dissolving 16.1 g of Na_2_HPO_4_·7H_2_O, 5.50 g of NaH_2_PO_4_·H_2_O, 0.75 g of KCl, and 0.246 g of MgSO_4_·7H_2_O in 800 mL of ultrapure water, adjusting the pH to 7.0, and diluting to 1 L). The resuspended samples underwent freeze–thaw cycles to lyse the cells. A mixture of 700 μL of Z‐buffer containing β‐mercaptoethanol (prepared by adding 0.27 mL of β‐mercaptoethanol to 100 mL of Z‐buffer) and 160 μL of dissolved ONPG solution (prepared by dissolving 0.008 g of ONPG powder in 2 mL of Z‐buffer and adjusting the pH to 7.0) was added to each control and experimental sample. The samples were incubated in a 30°C water bath for 10 min to allow the reaction to occur. The reaction was terminated by adding Na_2_CO_3_, and OD_420_ was measured to determine β‐galactosidase activity. The enzymatic activity was calculated using the formula.

$$\beta -\text{Galactosidase units}=1000\times {\mathrm{OD}}_{420}/\left(t\times V\times {\mathrm{OD}}_{600}\right)$$

$$t=10\;\min,V=0.1\;\mathrm{mL}\times \text{concentration factor}\left(5\right)$$

### 2.4 Bimolecular Fluorescence Complementation, BiFC

Two restriction sites, *SalI* and *BamHI*, were selected based on the MCS of the vector. Flower cDNA from *Arabidopsis* was used as a template for PCR amplification. After digestion with the selected restriction enzymes, the resulting fragments were ligated into the vector and transformed into *E. coli* DH5α cells. Plasmids were extracted from the transformed *E. coli* and subsequently introduced into *Agrobacterium tumefaciens* GV3101. The pSPYCE‐35S/PUC‐*AtSEP3*, pSPYCE‐35S/PUC‐*AtSEP3‐T5*, and pSPYNE‐35S/PUC‐*AtAP1* plasmids were transferred into *A. tumefaciens* and initially cultured in 10 mL of LB liquid medium. About 2–3 mL of the cultured solution was then added to 100 mL of LB medium containing with gentamicin, ampicillin, and rifampicin followed by further culture for 24‐36 h until the OD_600_ reached about 1.0.

The culture was centrifuged at 5000 rpm for 10 min, and the supernatant was discarded. About 6 mL of prepared penetrant (containing 4.264 g of MES, 0.408 g of MgCl_2_, dissolved in 200 mL of water, with 20 μL of 100 mmol/L acetosyringone dissolved in DMSO) was added to the bacterial pellet. The acetosyringone (AS) solution was diluted at in a 1:1000 ratio in the penetrant, and the bacterial pellet was gently resuspended. The suspension was incubated in the dark at 28°C for 3–4 h to facilitate preparation for subsequent experiments.

Subsequently, the bacterial solutions were mixed in a 1:1 ratio for use. The lower leaf epidermis of appropriately aged tobacco leaves was infiltrated with the bacterial mixture, labeled, and incubated in darkness for 24 h, followed by cultivation under normal light conditions for 48 h. After incubation, the infiltrated lower epidermis was excised and imaged using a laser confocal microscope (STELLARIS Cryo, Germany) to observe fluorescence signals. A 488‐nm argon laser line was employed for excitation, and fluorescence was detected using a 505–530 nm bandpass emission filter.

### 2.5 Model Prediction and Protein Docking

As the *Arabidopsis* *AtAP1* protein model was unavailable in the PDB database, the Protein Model Prediction Tool was utilized to generate a model for *AtAP1*. Predicted models of *AtAP1* and *AtSEP3* were subjected to docking studies using the Z‐Dock (https://zdock.wenglab.org/) and H‐Dock (http://Website.hdock.phys.hust.edu.cn/) platforms. The docking results are presented in Figure S1 and Tables S1 and S2.

### 2.6 Induced Expression of Soluble GST Tag Protein in *E. coli*

Three prokaryotic expression vectors were constructed to produce recombinant proteins *AtSEP3*, *AtSEP3‐T5*, and *AtSEP3‐DT5* in *E. coli* . After biotin labeling of probes, EMSA experiments were conducted to investigate the effect of leucine at position 116 on the interaction between *AtSEP3* and *AtAP1*. The kits used in this study included the GST‐tagged protein purification kit (P2262), EMSA probe biotin labeling Kit (GS008), and chemiluminescence EMSA kit (GS009), all purchased from Biyun Tian. The primary prokaryotic expression vector was pGEX‐4T‐3. *E. coli* DH5α strain was used for gene cloning and vector construction, whereas the *E. coli* BL‐21 (DE3) strain was utilized as host cells for the prokaryotic expression system. The primers used in the experiments are listed in Table 2.

**TABLE 2:** Primer sequences for construction of prokaryotic expression vectors.

Primer name	Sequences (5′ → 3′)
*SEP3‐*4T‐F	GCGGGATCCGGAAGAGGGAGAGTAGAATTGAAG
*SEP3*‐4T‐R	CGCGAATTCAATAGAGTTGGTGTCATAAGGTAAC
*AP1*‐probe‐F	TTTTCTAGGGCTT*CCATTTTTGG*ATTTTTTGATTAGCC
*AP1*‐probe‐R	GGCTAATCAAAAAATCCAAAAATGGAAGCCCTAGAAAA

### 2.7 Preparation of Transgenic Plants

The vector used was plant binary expression vector *pRI* 101‐AN was utilized in the study. The *E. coli* DH5α strain was employed for vector construction, and the *A. tumefaciens* GV3101 strain was used for genetic transformation. A single colony of *Agrobacterium* was selected and cultured in 10 mL of LB medium containing rifampicin (25 mg/mL), gentamicin (50 mg/mL), and kanamycin (50 mg/mL). The culture was centrifuged at 4000 rpm for 10 min at room temperature in a 50‐mL tube, and the bacterial solution's OD_600_ was adjusted to 1.0. A 400‐mL infiltration fluid was prepared with 0.866 g of 1/2 MS_0_ powder, 20 g of sucrose, and 400 mL of distilled water, adjusted to pH 5.7–5.8, with 200 μL of Silwet‐77 added. Site‐mutated *AtSEP3* was transformed into wild‐type *Arabidopsis* and the *sep3* mutant to examine the phenotypic effects of losing the interaction between *AtSEP3* and *AtAP1* on Arabidopsis growth and development. Wild‐type Col‐0, the s*ep3* mutant, *AtSEP3‐101* transgenic plants, and transgenic lines overexpressing *AtSEP3‐T5‐101* in both wild‐type and s*ep3* mutant backgrounds were cultivated in an artificial climate chamber with growth conditions set to 16‐h light/8‐h dark cycles at a temperature of 20°C–25°C.

## 3 Results

### 3.1 Yeast Two‐Hybrid (Y2H)

**FIGURE 3:**
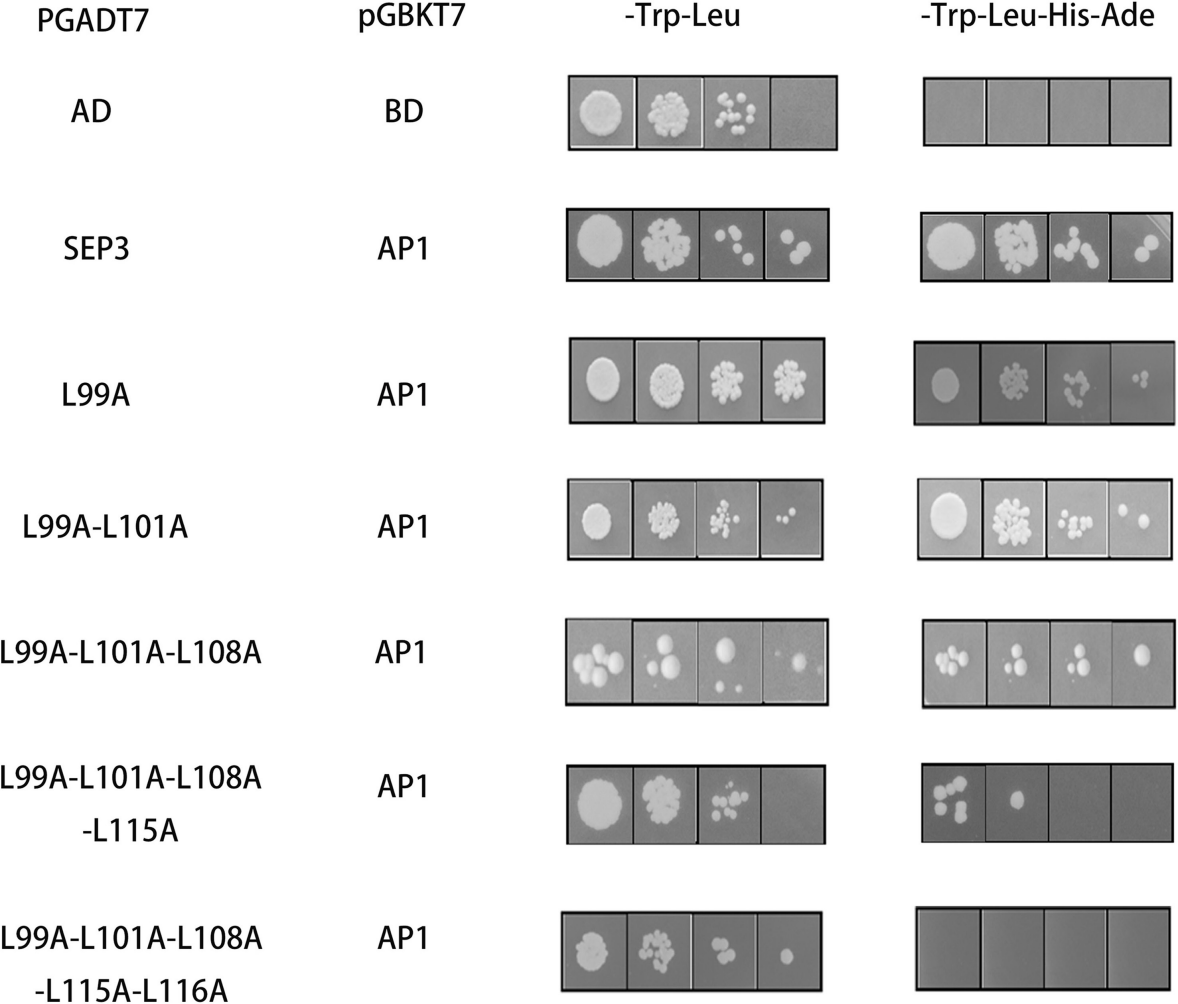
Yeast two‐hybrid diagram of *AtSEP3* carrying multipoint mutation and *AtAP1*. These mutations included single‐site mutations (Leu to Ala at position 99), double‐site mutations (Leu to Ala at positions 99 and 101), triple‐site mutations (Leu to Ala at positions 99, 101, and 108), four‐site mutations (Leu to Ala at positions 99, 101, 108, and 115), and five‐site mutations (Leu to Ala at positions 99, 101, 108, 115, and 116).

**FIGURE 4:**
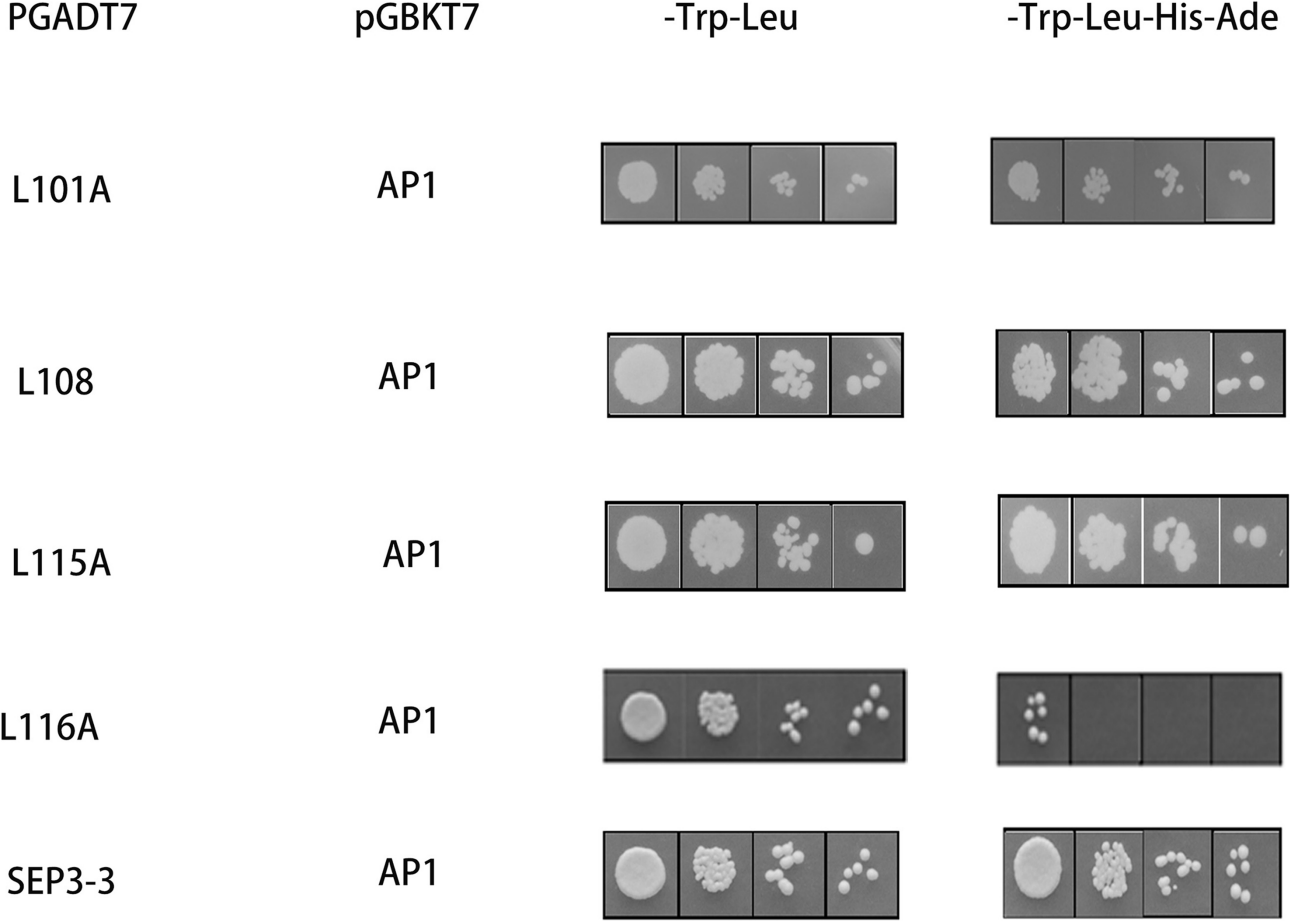
Yeast two‐hybrid diagram of *AtSEP3* carrying single point mutation, *AtSEP3.3* and *AtAP1*. These mutations included single‐site mutations (Leu to Ala at position 99, 101, 108, 115, and 116).

The constructed pGADT7‐*AtSEP3* vector and Arabidopsis *AtSEP3* mutants with single‐site and multiple‐site mutations were tested in a yeast two‐hybrid (Y2H) assay. As the number of mutation sites increased, the intensity of the interaction strength between *AtSEP3* and *AtAP1* was gradually decreased. When leucine at position 116 was mutated, *AtSEP3* completely lost its interaction capability with *AtAP1* (Figure 3). Additionally, separate mutations at leucines 99–116 revealed that mutating leucine 116 alone significantly reduced interaction strength (Figure 4).

### 3.2 β‐Galactosidase Activity

**FIGURE 5:**
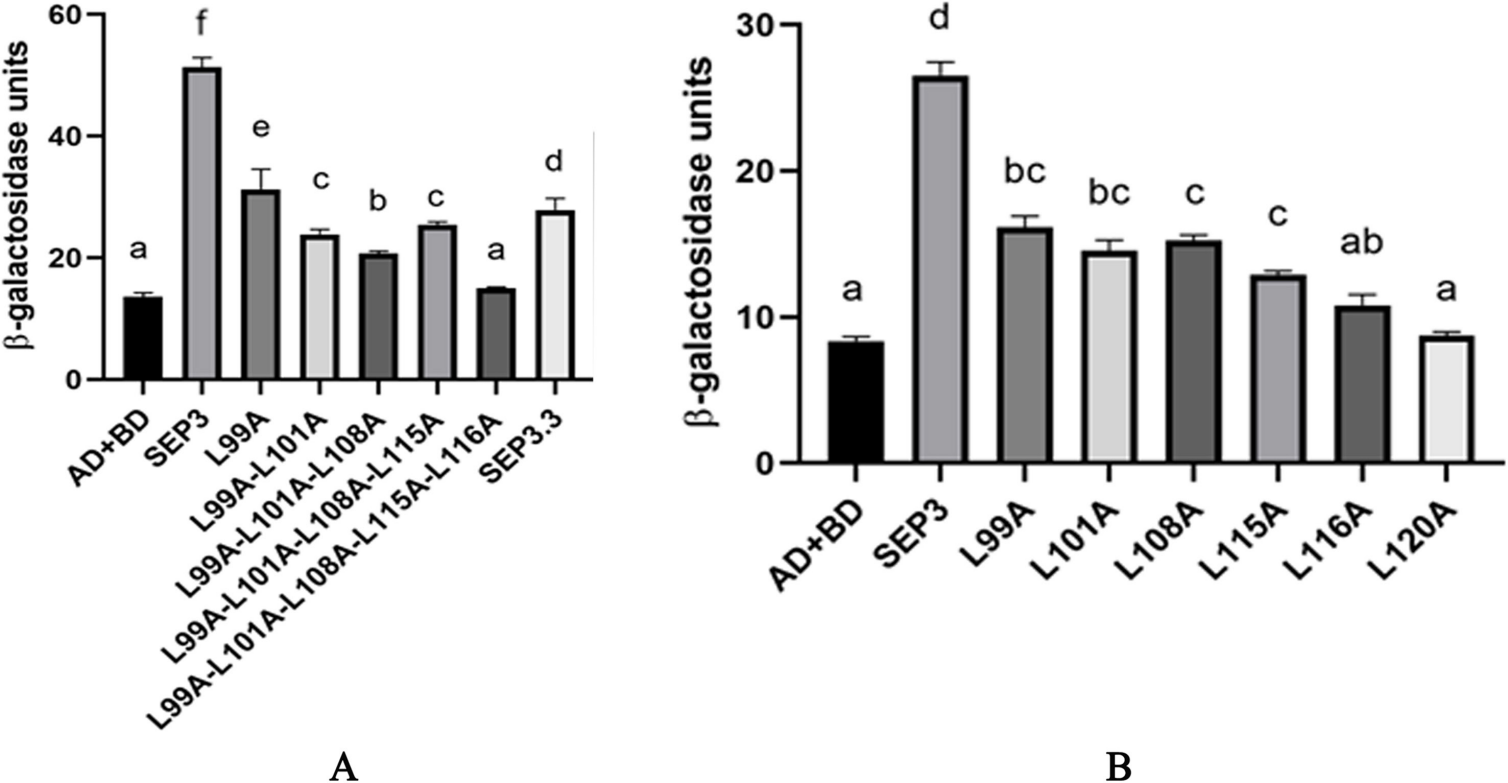
The galactosidase activity test results. (A) Interaction between *AtAP1* and *AtSEP3* carrying multipoint mutations, *AtSEP3.3*. (B) Interaction between *AtSEP3* carrying single point mutation and *AtAP1*.

The yeast colonies grown on the medium were selected and β‐galactosidase activity was measured. The results of this experiment were basically consistent with those of the results of Y2H assay. With an increasing number of leucine mutations, β‐galactosidase activity progressively decreased and was completely abolished when multiple mutations included the 116th leucine, matching the control group (ad + BD). The interaction between *AtSEP3.3*, a natural splicing variant of *Arabidopsis* and *AtAP1*, exhibited significantly reduced activity due to the absence of the K3 region (Figure 5A). In the single mutation experiments, activity was substantially decreased when the 116th leucine was mutated, comparable to the same as that of the control group (ad + BD). Previous studies have demonstrated the importance of the 120th leucine in SEP3–protein interactions; this study corroborated these findings, showing consistent results (Figure 5B).

### 3.3 BiFC

**FIGURE 6:**
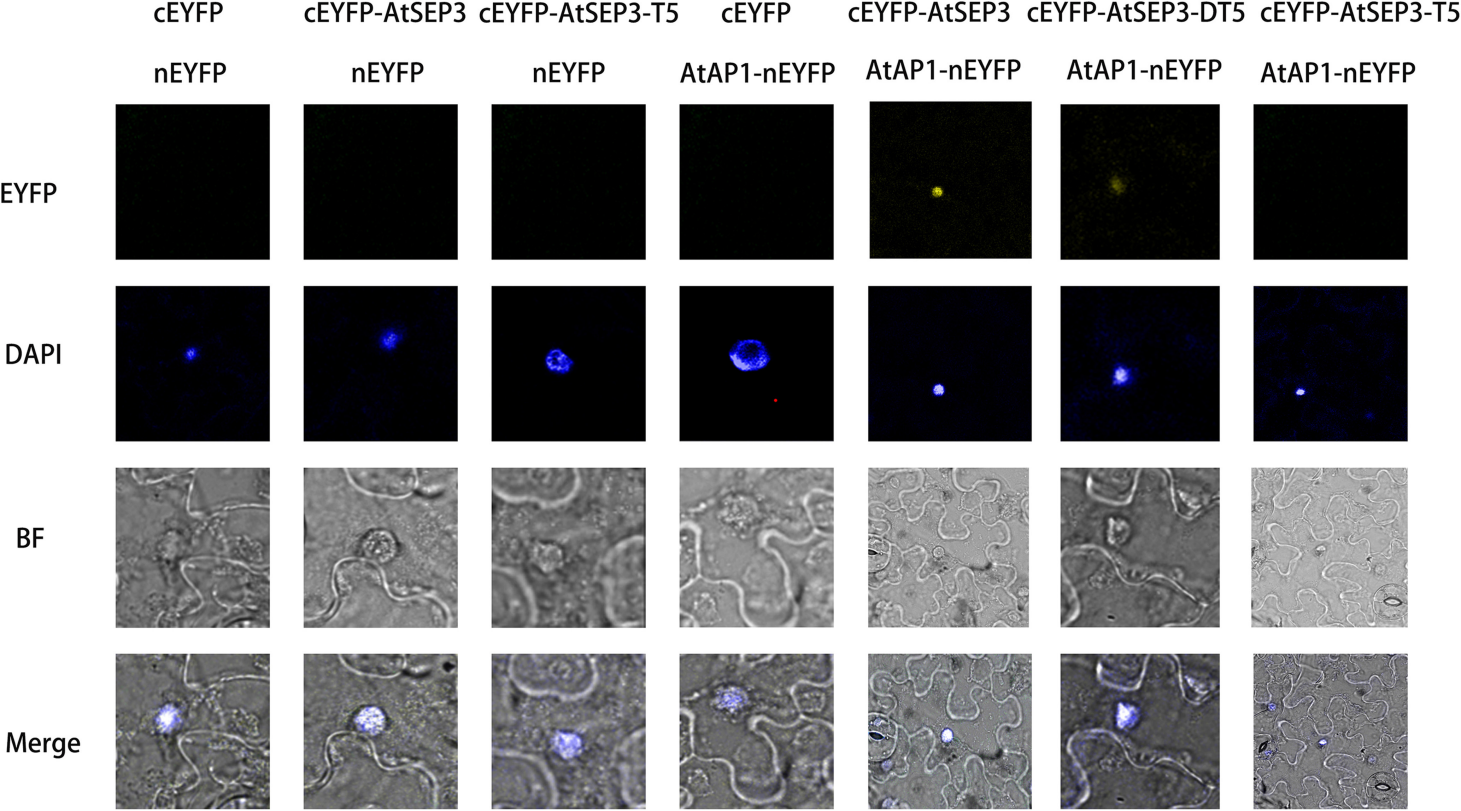
Fluorescence complementation (BiFC) assay of the interaction between the proteins encoded by *AtSEP3*, *AtSEP3‐T5*, and *AtAP1*.

No fluorescence signal was detected between *AtSEP3‐T5* and *AtAP1*, aligning with the results of Y2H results. The fluorescence signal between *AtSEP3‐DT5* and *AtAP1*, which involved a single mutation of the 116th leucine alone, was markedly weakened, which was also consistent with the above Y2H observations (Figure 6).

### 3.4 Prediction of Binding Sites and Chemiluminescent EMSA

**FIGURE 7:**
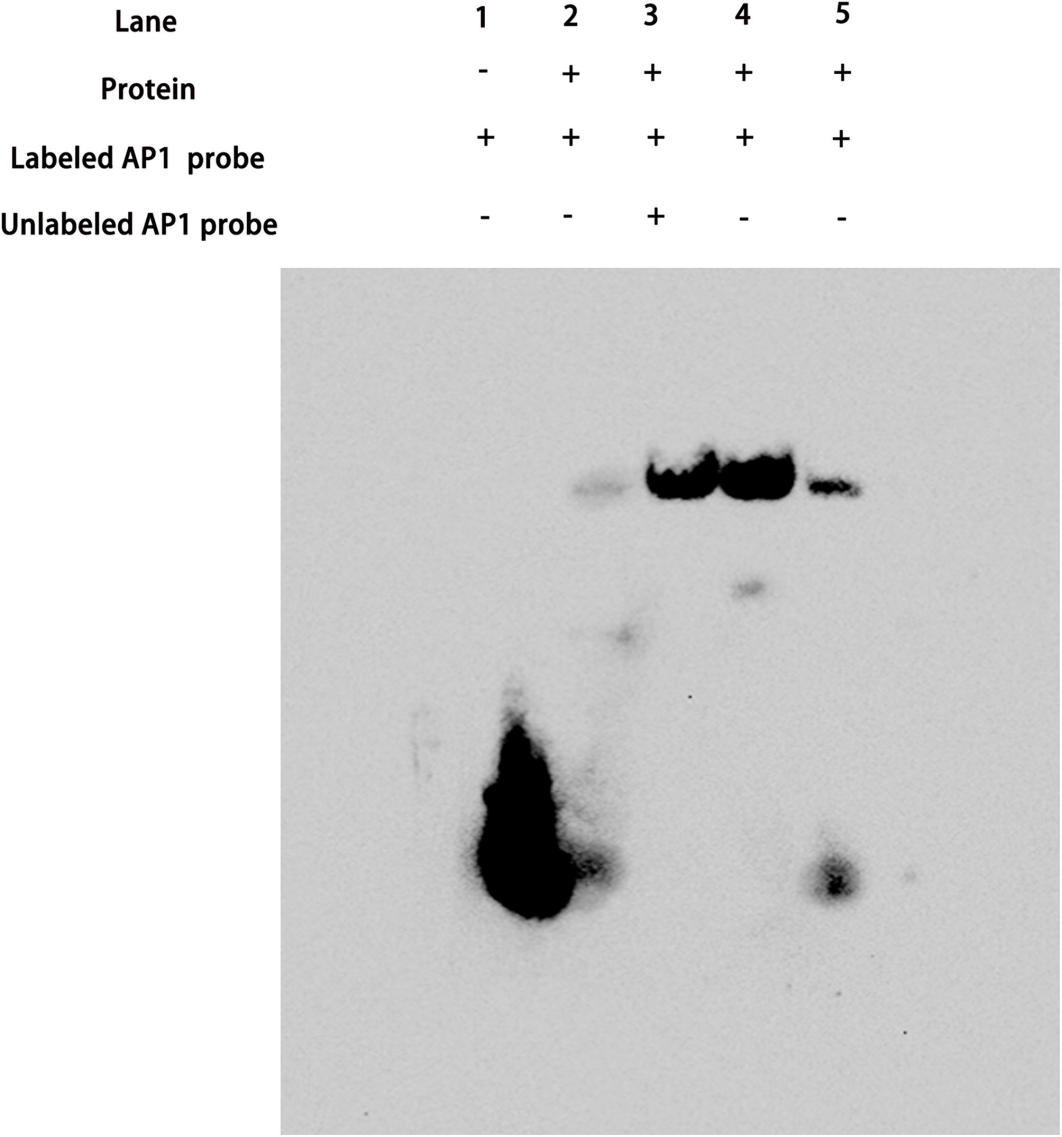
EMSA results of DNA probe binding to *AtSEP3*, *AtSEP3‐DT5*, and *AtSEP3‐T5*. The purified protein was bound to the probe (TTTTCTAGGGCTTCCATTTTTGGATT TTTTGATTAGCC) at room temperature. The first lane is the blank probe, the second lane is the binding band of the labeled probe to the mutant SEP3‐T5 protein, the third lane incorporates the wild‐type SEP3 protein and the labeled and unlabeled AP1 probes, the fourth lane is the dimerization band formed by the binding of the wild‐type SEP3 protein to the labeled AP1 probe, and the fifth lane is the binding band of the SEP3‐T5 mutant protein to the labeled AP1 probe.

As illustrated in Figure 7, the first lane represents a blank probe, and the second lane demonstrates that the AP1 probe of the single CArG cassette binds to SEP3 with a weaker binding band and fewer dimer bands. The third lane shows a lower density of binding bands compared to the fourth lane, indicating that the binding is specific. The fourth lane displays a higher density of binding bands. The fifth lane reveals a significantly reduced density compared to the wild‐type binding band, with a notable decrease in the ability to bind and form dimers. Mutation of five consecutive LEUs severely affects the ability of SEP3 to form dimers. A single mutation at the 116th Leu also results in a substantial reduction in binding ability. This effect may be due to the disruption of the leucine zipper, which impairs the interaction between the two proteins and thus reduces the ability to bind to the DNA probe. The fifth lane shows a much lower density in comparison to the wild‐type binding band, with a considerable decrease in the ability to bind and form dimers. The dimer formation capacity of SEP3 is significantly impaired when five consecutive LEUs are mutated. Mutation of the 116th Leu alone also greatly reduces binding ability, likely due to the disruption of the leucine zipper, affecting the interaction between the proteins and weakening the ability to bind to the DNA probe.

### 3.5 Phenotypical Analysis of Transgenic Plants

**FIGURE 8:**
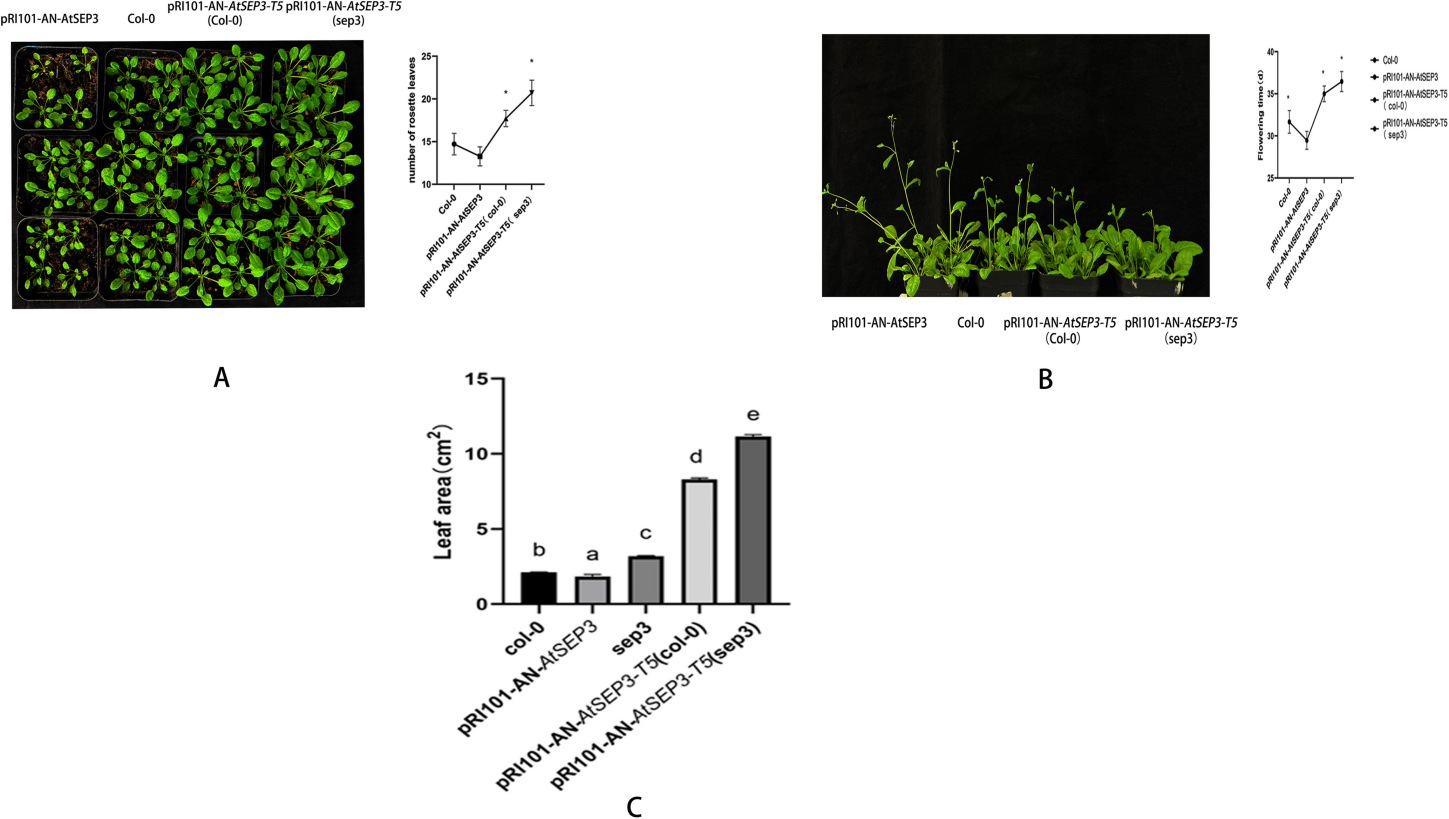
Phenotypic observation. (A) Phenotype and quantitative statistics of rosette leaves in *AtSEP3*, *AtSEP3‐T5* (Col‐0), and *AtSEP3‐T5* (*sep3*) transgenic plants. (B) Bolting situation of *AtSEP3*, *AtSEP3‐T5* (Col‐0), and *AtSEP3‐T5* (*sep3*) transgenic plants. (C) Statistics analysis of leaf area in *AtSEP3*, *AtSEP3‐T5* (Col‐0), and *AtSEP3‐T5* (*sep3*) transgenic plants.

*AtSEP3* and *AtAP1* are key factors in the floral transition of *Arabidopsis*. In this study, transgenic plants of *AtSEP3*, *AtSEP3‐T5* (Col‐0), and *AtSEP3‐T5* (*sep3*) were developed (Table 3). After the plants grew four true leaves, they were transplanted into the soil. After 4–5 weeks of growth, the rosette leaves was photographed before bolting, and the average number of rosette leaves per line was determined. *AtSEP3‐T5* (Col‐0) and *AtSEP3‐T5* (*sep3*) exhibited a significantly higher number of rosette leaves compared to wild‐type plants, whereas *AtSEP3* overexpression transgenic plants had the fewest rosette leaves (Figure 8A).

**TABLE 3:** Primers for the acquisition and identification of transgenic plants.

Primer name	Sequences (5′ → 3′)
*SEP3*‐F‐101	GCGCATATGGGAAGAGGGAGAGTAGAATTG
*SEP3*‐R‐101	CGCGAATTCAATAGAGTTGGTGTCATAAGG
pRI101‐AN‐NOST‐R	ATCGCAAGACCGGCAACAGG
Wis109_05E‐LP	GGTCCAAGATCTTCTCCCAAC
Wis109_05E‐RP	ATTTGGGATACCCCAAATCTG
LbP1	TGATCCATGTAGATTTCCCGGACATGAAG

In agreement with the observations of rosette leaf growth, *AtSEP3* plants flowered earlier and had a shorter vegetative growth period. By contrast, *AtSEP3‐T5* (Col‐0) and *AtSEP3‐T5* (*sep3*) transgenic plants bolted later and had a longer vegetative growth period compared to both wild‐type and *AtSEP3* transgenic plants (Figure 8B). The leaf area of each line was also measured, and the results revealed that *AtSEP3‐T5* (Col‐0) and *AtSEP3‐T5* (*sep3*) transgenic plants had significantly larger leaf areas compared to Col‐0 wild‐type plants (Figure 8C).

**FIGURE 9:**
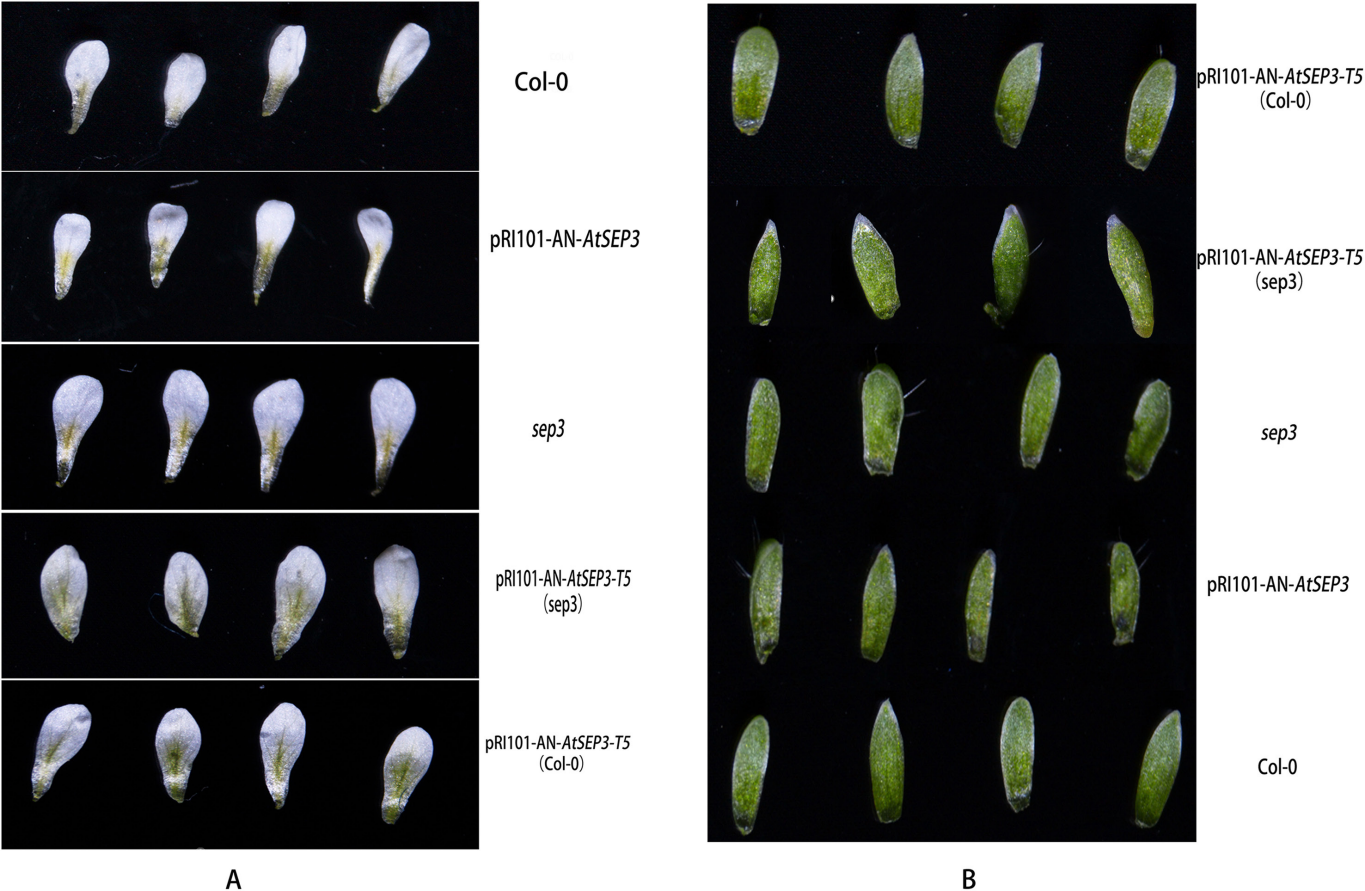
Phenotypes of the first and second whorl floral organs. (A) Petals in Col‐0,*AtSEP3*, *AtSEP3‐T5* (Col‐0), and *AtSEP3‐T5* (*sep3*) transgenic plants. (B) Sepals in Col‐0, *AtSEP3*, *AtSEP3‐T5* (Col‐0), and *AtSEP3‐T5* (*sep3*) transgenic plants.

**FIGURE 10:**
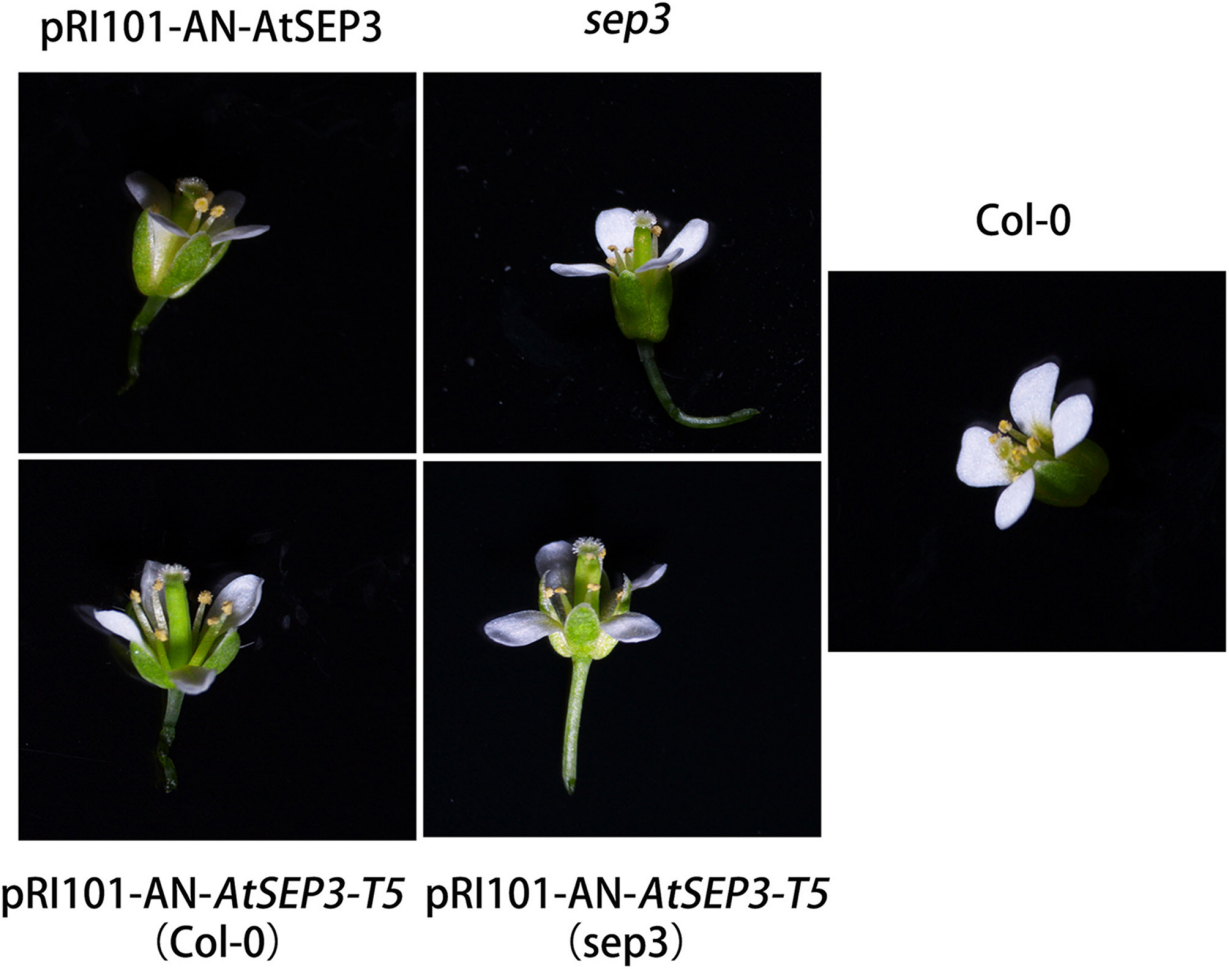
Flower phenotypes of Col‐0, *AtSEP3*, *AtSEP3‐T5* (Col‐0), and *AtSEP3‐T5* (*sep3*) transgenic plants.

Given that *AtSEP3* and *AtAP1* are also involved in sepal and petal development, the petals and sepals opened at full flowering stage were examined under a microscope in this work. No significant differences were observed between the *AtSEP3* transgenic plants and the wild‐type Col‐0. However, compared to *AtSEP3* transgenic plants and wild‐type *Arabidopsis*, *sep3* mutants had larger petals. The petals of *AtSEP3‐T5* (Col‐0) and *AtSEP3‐T5* (*sep3*) transgenic plants were not only larger than those of *AtSEP3* transgenic plants and wild‐type plants but also exhibited sepal‐like green tissue on the petals, especially along the leaf veins (Figure 9A). Additionally, a small petal‐like structure appeared along the edges of the sepals in *AtSEP3‐T5* (*sep3*) transgenic plants (Figure 9B). Furthermore, the flowers of *AtSEP3‐T5* (Col‐0) and *AtSEP3‐T5* (*sep3*) transgenic plants were notably larger (Figure 10).

## 4 Discussion

The transition from vegetative to reproductive growth in plants is governed by the coordinated action of transcription factors that integrate environmental factors, such as photoperiod, temperature, and nutritional status, as well as internal signals like plant age. Over the past three decades, various transcription factors involved in regulating the flowering transition and floral organ development have been identified in *Arabidopsis* (Rümpler et al. 2018; Bao et al. 2020; Ayra et al. 2021   . The MADS‐box protein FLC, a major repressor of flowering, is epigenetically downregulated during vernalization, but the closely related protein FLM mediates the plant's response to temperature changes (Martínez‐Gómez et al. 2021). SVP, another MADS‐box protein, forms a complex with FLC and FLM‐β, which is involved in the plant's response to low and high temperatures, respectively (Hnisz et al. 2013; Xuan et al. 2022; Zong et al. 2022). In combination with the MADS proteins SOC1 and AGL24 (Chang et al. 2022), which are positive regulators of flowering time, SVP also plays a role in the later stages of floral meristem development. The floral meristem identity genes *LEAFY (LFY)* (Surkova and Samsonova 2022) and *APETALA1 (AP1)* (Yamaguchi 2021) promote each other's expression in a positive feedback loop (Liang et al. 2019), thereby reinforcing floral meristem organization. AP1 and LFY can activate floral homoeologous heterozygous genes (Winter et al. 2011), which then function together to determine the identity of the four floral organs: sepals, petals, stamens, and carpels. According to the tetrameric model of flower development, floral homologous heterotrimers form organ‐specific tetrameric protein complexes, with SEPALLATA proteins (including SEP1, SEP2, SEP3, and SEP4) mediating this higher order interaction with overlapping functions (Martínez‐Gómez et al. 2021).

To investigate the role of the leucine zipper in the SEP3 K1 region of *Arabidopsis* and its interaction with AP1, along with the impact of this interaction of two proteins on the growth and development of *Arabidopsis*, a protein docking model was employed to analyze the leucine residues associated with the interaction between SEP3 K1 region and AP1. Site‐directed mutagenesis was performed on leucine residues in the SEP3 K1 region, targeting single and multiple sites, and the interactions between molecules were analyzed using Y2H assays, bimolecular fluorescence complementation, and model prediction. The findings indicate that leucine at position116 is particularly critical for the interaction between SEP3 and AP1. In the study, wild‐type SEP3, SEP3‐T5, and SEP3‐DT5 proteins were further purified, and their binding to a labeled AP1 probe was analyzed using EMSA. Results revealed that after mutating five consecutive leucine residues, the binding affinity between SEP3 and the AP1 probe was significantly reduced. A single mutation at leucine 116 also resulted in a notable decrease in binding ability compared to the wild type, despite some residual interaction. Furthermore, the regulatory effects of the SEP3‐AP1 interaction on the growth and development of *A. thaliana* were analyzed. When the interaction between these proteins was abolished, the vegetative growth phase of *Arabidopsis* was extended, floral organ development became more robust compared to the wild type, the petals developed a green, sepal‐like structure, and subtle alterations were observed in sepal development.

**FIGURE 11:**
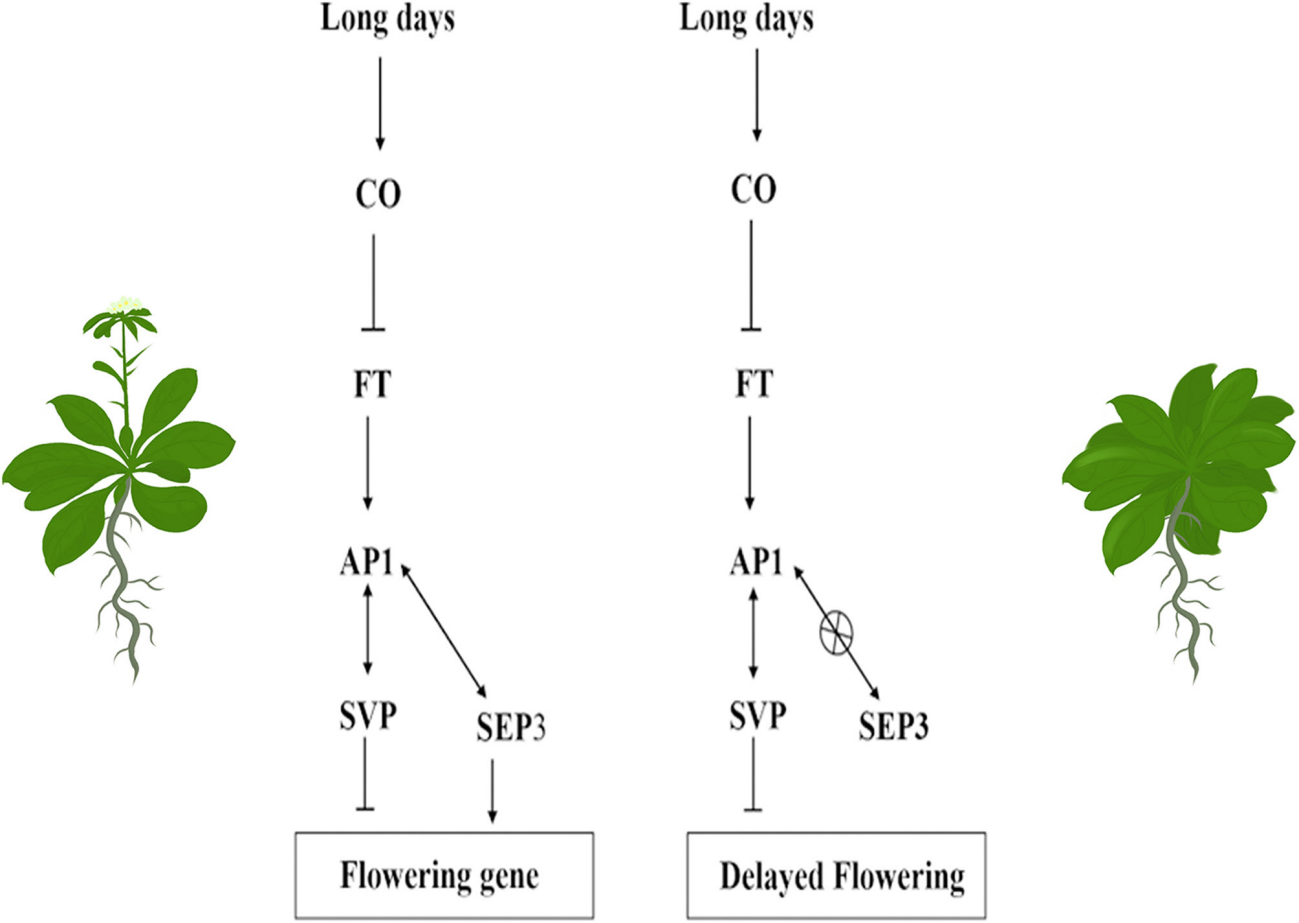
Diagram of flowering induction pathway in *Arabidopsis*.

In summary, leucine 116 of SEP3 is crucial for its interaction with AP1. Loss of this interaction due to mutation results in *Arabidopsis* exhibiting an extended vegetative growth phase, delayed flowering, and enhanced development of rosette leaves and floral organs (Figure 11). The absence of interaction also leads to the formation of sepaloid structures in petals, with minimal impact on sepal development, limited to subtle changes at the edges. This study offers a novel approach to investigating the interaction mechanism between AP1 and SEP3.

## Author Contributions

**Xiao‐Min Tan:** investigation. **Ya‐Ru Li:** investigation. **Man‐Ru Song:** investigation. **Ling‐Na Yuan:** investigation. **Ye Liu:** investigation. **Zi‐Xin Zhao:** investigation. **Qi Meng:** investigation. **Xuan Huang:** data curation, formal analysis. **Ye‐Ye Ma:** visualization, methodology. **Zi‐Qin Xu:** conceptualization, writing.

## Conflicts of Interest

The authors declare no conflicts of interest.

## Peer Review

The peer review history for this article is available in the Supporting Information for this article.

## Supporting information


**Data S1** Peer Review.

**Table S1** Interaction binding energy table of two proteins after docking.

**Table S2** Receptor‐ligand interface residue pair(s).

**Figure S1** Supporting Information.
