# Supplementary figures and images for: The Molecular Mechanism of Interaction Between SEPALLATA3 and APETALA1 in Arabidopsis thaliana
Source: Plant Direct. 2025 Mar 30;9(4):e70052. doi: 10.1002/pld3.70052 (PMC11955279; doi:10.1002/pld3.70052)

Supplementary table1 Interaction binding energy table of two proteins after docking


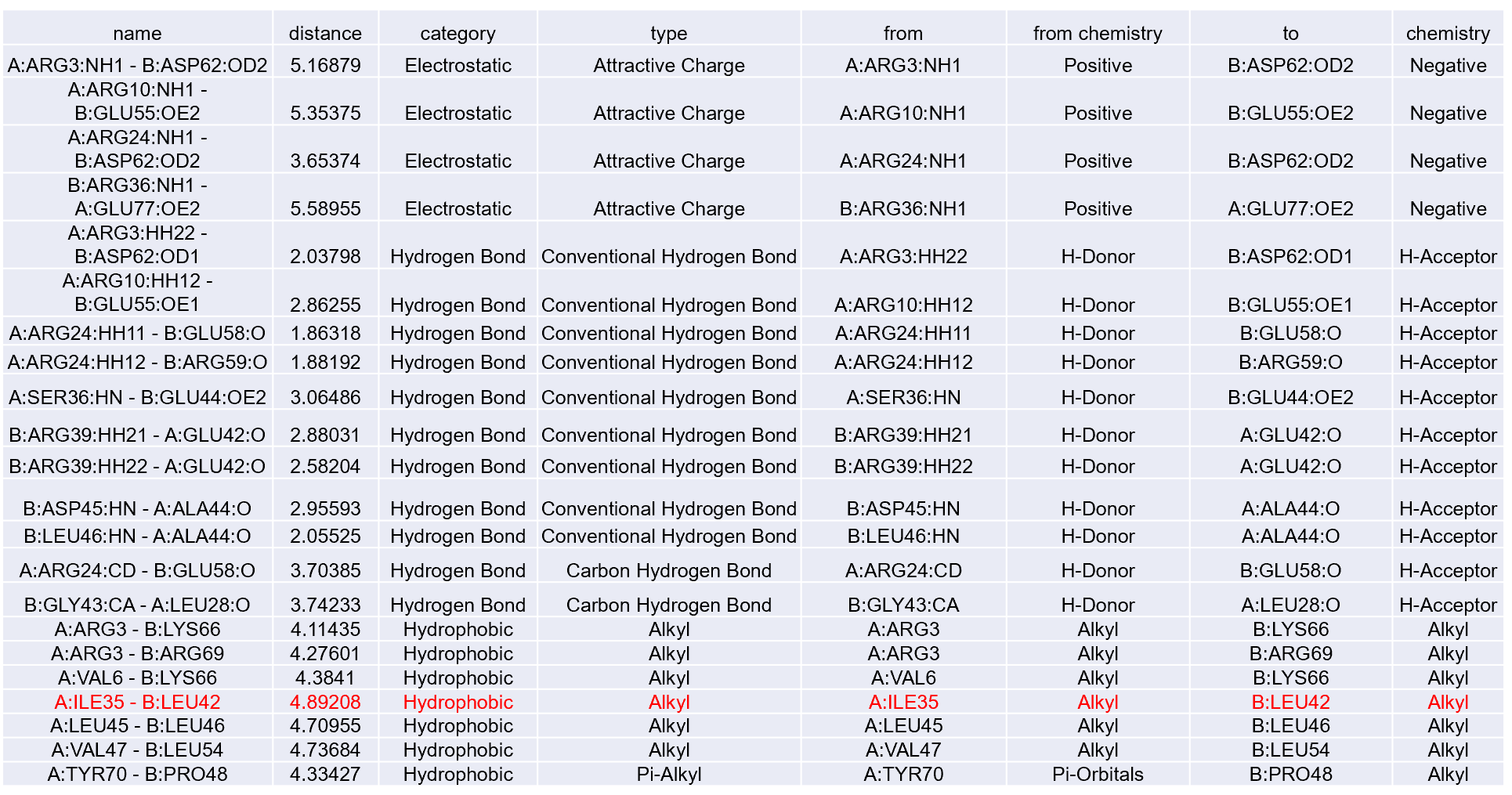

Supplement: Supplementary file 2 — Table S1 Interaction binding energy table of two proteins after docking. [file PLD3-9-e70052-s003.docx]

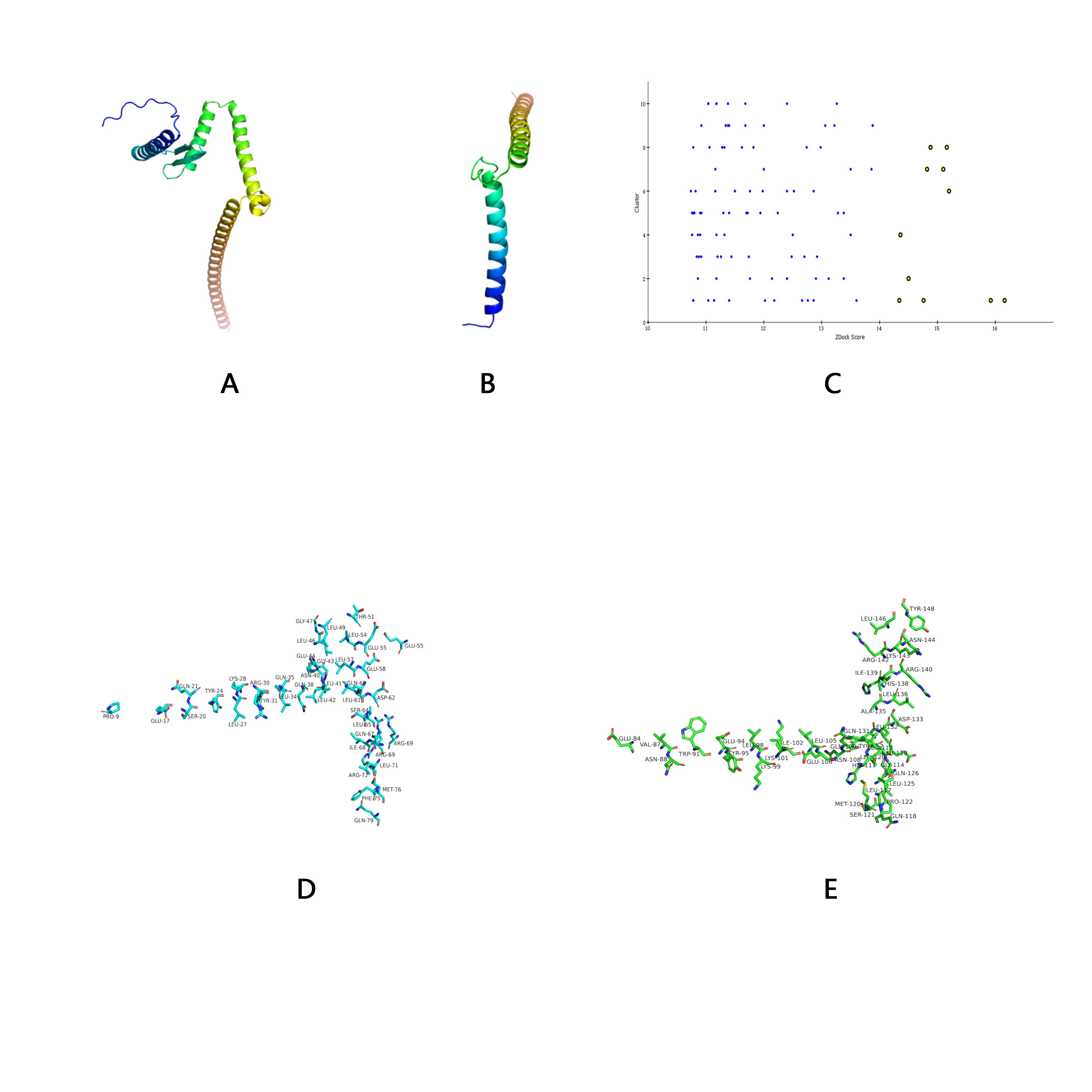

Supplement: Supplementary file 4 — Figure S1 Supporting Information. [file PLD3-9-e70052-s002.jpg]
