# Supplementary material for: The Molecular Mechanism of Interaction Between SEPALLATA3 and APETALA1 in Arabidopsis thaliana
Source: Plant Direct. 2025 Mar 30;9(4):e70052. doi: 10.1002/pld3.70052 (PMC11955279; doi:10.1002/pld3.70052)
Supplement: Supplementary file 3 — Table S2 Receptor‐ligand interface residue pair(s). [file PLD3-9-e70052-s001.docx]

Supplementary table 2 Receptor-ligand interface residue pair(s)

| Receptor interface residue(s) | Ligand interface residue(s) | binding energy | Receptor interface residue(s) | Ligand interface residue(s) | binding energy |
| --- | --- | --- | --- | --- | --- |
| 84A | 9A | 3.56 | 101A | 31A | 0.995 |
| 87A | 17A | 1.904 | 101A | 35A | 4.041 |
| 87A | 21A | 4.975 | 102A | 27A | 3.127 |
| 88A | 17A | 3.983 | 102A | 30A | 4.923 |
| 91A | 17A | 2.513 | 102A | 31A | 2.902 |
| 91A | 20A | 3.823 | 102A | 34A | 3.533 |
| 91A | 21A | 2.933 | 105A | 31A | 3.608 |
| 91A | 24A | 3.234 | 105A | 34A | 3.191 |
| 94A | 24A | 3.171 | 105A | 35A | 3.246 |
| 95A | 24A | 3.340 | 105A | 38A | 3.506 |
| 95A | 27A | 3.529 | 106A | 30A | 4.495 |
| 98A | 24A | 3.921 | 106A | 34A | 3.545 |
| 98A | 27A | 3.942 | 108A | 38A | 3.365 |
| 98A | 28A | 3.552 | 109A | 34A | 3.685 |
| 98A | 31A | 3.011 | 109A | 38A | 4.048 |
| 99A | 27A | 4.485 | 109A | 41A | 4.715 |
| 111A | 68A | 3.116 | 111A | 64A | 4.368 |
| 112A | 38A | 3.165 | 126A | 72A | 3.641 |
| 112A | 41A | 3.942 | 128A | 68A | 3.294 |
| 112A | 42A | 3.140 | 129A | 65A | 3.457 |
| 112A | 60A | 4.949 | 129A | 68A | 4.546 |
| 112A | 64A | 1.602 | 129A | 69A | 1.341 |
| 112A | 68A | 4.458 | 129A | 72A | 2.842 |
| 113A | 60A | 4.516 | 131A | 42A | 3.895 |
| 113A | 64A | 3.742 | 132A | 61A | 3.891 |
| 114A | 64A | 4.135 | 132A | 64A | 3.624 |
| 114A | 67A | 3.971 | 132A | 65A | 3.752 |
| 114A | 68A | 4.280 | 132A | 68A | 3.416 |
| 117A | 67A | 4.706 | 133A | 65A | 2.468 |
| 117A | 68A | 3.206 | 133A | 69A | 2.613 |
| 117A | 71A | 2.470 | 135A | 41A | 4.412 |
| 118A | 71A | 4.368 | 135A | 42A | 2.232 |
| 118A | 75A | 4.345 | 135A | 43A | 3.523 |
| 120A | 75A | 4.140 | 135A | 61A | 4.361 |
| 121A | 75A | 4.849 | 136A | 58A | 3.361 |
| 122A | 72A | 3.533 | 136A | 61A | 4.690 |
| 122A | 75A | 3.386 | 136A | 62A | 3.909 |
| 122A | 76A | 4.911 | 136A | 65A | 4.526 |
| 122A | 79A | 3.631 | 138A | 42A | 4.731 |
| 125A | 68A | 4.073 | 138A | 43A | 3.420 |
| 125A | 71A | 4.779 | 138A | 44A | 4.432 |
| 125A | 72A | 3.630 | 138A | 46A | 4.112 |
| 125A | 75A | 4.771 | 139A | 40A | 4.122 |
| 139A | 43A | 4.420 | 142A | 54A | 4.348 |
| 139A | 46A | 3.769 | 143A | 51A | 4.815 |
| 139A | 54A | 3.527 | 143A | 54A | 3.654 |
| 139A | 57A | 3.410 | 143A | 55A | 2.387 |
| 139A | 58A | 3.991 | 143A | 58A | 3.912 |
| 139A | 61A | 2.901 | 140A | 58A | 3.205 |
| 146A | 49A | 4.676 | 142A | 46A | 3.938 |
| 146A | 54A | 3.854 | 142A | 47A | 3.413 |
