# Supplementary material for: The Molecular Mechanism of Interaction Between SEPALLATA3 and APETALA1 in Arabidopsis thaliana
Source: Plant Direct. 2025 Mar 30;9(4):e70052. doi: 10.1002/pld3.70052 (PMC11955279; doi:10.1002/pld3.70052)
Supplement: Supplementary file 1 — Data S1 Peer Review. [file PLD3-9-e70052-s004.pdf]

Plant Direct Open Access

The Molecular Mechanism of Interaction between AtSEP3 and AtAP1 in *Arabidopsis thaliana*

Running Title： Interactions Between AtSEP3 and AtAP1 in Arabidopsis thaliana

Xiao-Min Tan, Ya-Ru Li, Man-Ru Song, Ling-Na Yuan, Zi-Xin Zhao, Ye Liu, Qi Meng, Xuan Huang, Ye-Ye Ma*, Zi-Qin Xu*

Decision Letter Round 1:

October 30, 2024

Dr. Ziqin Xu
Northwest University
Institute of Life Science
Taibaibeilu 229#
Xian 710069
China

RE: The Molecular Mechanism of Interaction between AtSEP3 and AtAP1 in Arabidopsis thaliana

Dear Dr. Xu:

Thank you for submitting to Plant Direct. All required reviews have been returned and we have now finished our evaluation of your manuscript. In light of the reviewers' and editor's comments, further revisions are needed before the paper can be accepted for publication in Plant Direct.

Please view the editors' and reviewers' comments below and use their suggestions as a guide while you work on your revision.

When uploading the revised version of this article, please be sure to include the following:

-A word document that contains your response to the reviewers. You should respond to each reviewer comment and note the changes made to the manuscript. If you do not agree with a reviewer's comment and choose not to make a suggested revision, please explain why. Please try to provide as complete an answer as possible to each reviewer's criticisms in the "response to reviewers" section.
-A tracked changes document
- A clean version of the latest version of the manuscript

Plant Direct
Open Access

To upload your revision, please click the link below.
https://plantdirect.msubmit.net/cgi-bin/main.plex?el=A3Lr6jz1A2DFa2I3A9ftd4lCeGjD0BVwk8Dd2LTA4hwZ

In order to provide as timely a service as possible, we ask that your revision is resubmitted within three months after receipt of this request. If an extension is needed, please send a request, along with a brief explanation, to the editorial office at plantdirect@wiley.com .

Thank you very much for giving us an opportunity to review your work. I look forward to receiving the next version.

Sincerely,
Yan Lu

--- Editor comments:

--- Reviewer comments:
Reviewer #2:

The interaction among different MADS transcriptional factors are important for flower development. In this paper, Tan and colleagues predicted the key residues of SEP3 for SEP3 and AP1 interaction. Through Y2H, BiFC and EMSA assays, they found the 116th residue of SEP3 is important. Understanding the detailed interaction mechanism between SEP3 and AP1 is interesting. However, many concerns need to be addressed to improve this work.

Major revisions:
1. Since this manuscript is focused on the sites of SEP3 for SEP3 and AP1 interaction, the authors should provide the information about AP1, SEP3 and their relationship in the Introduction section, including the phenotype of their mutants (especially sepal and petal phenotypes) and the importance of SEP3-AP1 complex for flower development.
2. Line 186-191: the authors found that the 115TH and 116th residues of SEP3 are important for AtSEP3 and AP1 interaction through D-dock and H-dock websites. This prediction is the prerequisite for this

paper. However, the detailed prediction process is lacked. Is it predicted by the structure of protein interaction? Could the authors provide the predicted structure of AtSEP3 and AP1 interaction? Please include the website link of D-dock and H-dock websites.
3. Line 240-246: why the authors chose the 99th, 101th and 108th residues of SEP3 to mutate as the control? This was not mentioned in the Materials and Methods section nor in the Results section. In addition, Fig. 1, 2 and 4 were also not present in the main text. What does SEP3-3 mean in Fig. 4? I also can't find any data to support the conclusion (AtAP1 and ATAP1 interaction was gradually weakened) at line 242.
4. Due to the redundancy of SEP genes, the abnormal phenotype of single sep mutant is subtle. It's better if the authors would try whether the other three SEPs (SEP1, SEP2 and SEP4) could interact with AP1, and if yes, whether the corresponding 116th residue of SEP3 in SEP1, SEP2 and SEP4 is also important?
5. Besides the AP3/PI/SEP3/AP1 complex, SEP3 also can form complexes with AP3, PI and AG (Theissen et al., 2016; Ruelens et al., 2017). Both complexes are important for regulating petal and stamen development. So whether the 116th residues of SEP3 is also essential for AP3/PI/SEP3/AG complex formation?

Minor:
1. Many lines lack the corresponding references, like line 86 "AP1 and AP2 for B-class gene activation", line 191 "confirming earlier findings", lines 257-258 "previous studies show the 120th leucine", line 292 "the function of SEP3 and AP1 for floral transition". Please check out through the manuscript.
2. Line 83: replace ABCDE model with ABCE model, because D-class genes are only confirmed in Arabidopsis and petunia.
3. I can't find the Table information (line 189 Table 2.2 and line 191 Table 2.3) in the merged manuscript or any Supplemental data.
4. Line 211-212: lack the detailed information about "AtSEP3, AtSEP3-T5 and ATSEP3-DT5". Line 268: no information was provided for AtSEP3-T5. In addition, all figure legends are too brief to understand the figure for us.
5. The sentences in lines 263-267 should be presented in the Material and Methods and the sentences in lines 275- 281 should be presented in the figure legends.

Reviewer #3:

The experimental design of your study is robust, and the research aims are clearly articulated in the abstract. However, from the Materials and Methods section onward, I observed several gaps in essential details. For example, the tobacco plants used for BiFC assays are not mentioned in the Plant Material section; solution concentrations are missing; protein extraction protocols are not included; and the number of plants analyzed for the phenotypic assessment is not specified. This lack of information makes it challenging to thoroughly evaluate the work.

Please review the attached document, where I have provided comments that may be useful.

Additionally, a key flowering gene, Flowering locus T (FT), is not addressed anywhere in the paper, either in the introduction or the analysis. This omission is concerning, as it is critical to consider FT levels when reporting early flowering in a transgenic line. I strongly recommend performing qPCR analysis on FT and its repressor TOPLESS (TPL) and COSTANS (CO) to support the observed phenotype.

Decision Letter Round 2:

February 6, 2025
Dr. Ziqin Xu
Northwest University
Institute of Life Science
Taibaibeilu 229#
Xian 710069
China

MSID: 2024-01598-TWR1
MS TITLE: The Molecular Mechanism of Interaction between AtSEP3 and AtAP1 in Arabidopsis thaliana

Dear Dr. Ziqin Xu:

I am pleased to inform you that your manuscript "The Molecular Mechanism of Interaction between AtSEP3 and AtAP1 in Arabidopsis thaliana" has been accepted for publication in Plant Direct.

Your article will appear online in the next available issue of Plant Direct. To ensure your article gets published as quickly as possible, please pay attention to the steps detailed below. We have found that most of the delays happen at this stage, especially at the payment stage, so please respond as quickly as possible when prompted.

License Agreement: Once your article has been accepted it will move to Production and undergo admin and file checking - you may receive an email with any queries we have at this stage. When all required items are received by the publisher and queries resolved, the corresponding author will receive an email from Wiley's Author Services system which will ask them to log on at https://authorservices.wiley.com/bauthor and will present them with the appropriate license for completion. Your article cannot be published until both the signed license agreement and payment of the article fee have been received.

Payment of the Open Access Article Publication Fee: All articles published in Plant Direct are fully open

access: immediately and freely available to read, download and share. Plant Direct charges a publication fee to cover the publication costs. The corresponding author for this manuscript should have already received a quote from the payments team (cs-openaccess@wiley.com) with the estimated article publication fee; please email cs-openaccess@wiley.com if this has not been received. The corresponding author should log on to the Wiley Author Services site, where the publication fee can be paid by credit card or an invoice. Pro Forma can also be requested. Payment of the publication charge must be received before the article will be published online.

Proofs: You will have the opportunity to look over your paper once more when you receive the author proofs for your article. The proofs will be with you in approximately two weeks. Please note that, in addition to publishing reviewer comments, the author's responses to review comments will also be published alongside the final version of the paper. If you would not like the author's responses to be published, please contact the editorial office at plantdirect@wiley.com .

Promotion of your article: You can help your research get the attention it deserves! Check out Wiley's free Promotion Guide for best-practice recommendations for promoting your work at www.wileyauthors.com/eeo/guide. And learn more about Wiley Editing Services which offers professional video, design, and writing services to create shareable video abstracts, infographics, conference posters, lay summaries, and research news stories for your research at www.wileyauthors.com/eeo/promotion.

Thank you again for your contribution to Plant Direct. If you have any questions, feel free to contact the editorial office at plantdirect@wiley.com .

Sincerely,

Yan Lu

Gustavo MacIntosh

Editor, Plant Direct

Response to Reviewer:

Reviewer #2:

Major revisions:

Comment 1: Since this manuscript is focused on the sites of *SEP3* for SEP3 and AP1 interaction, the authors should provide the information about AP1, SEP3 and their relationship in the Introduction section, including the phenotype of their mutants (especially sepal and petal phenotypes) and the importance of SEP3-AP1 complex for flower development.

Response: Thank you for the valuable feedback on our manuscript. With regard to your comments, we have made improvements, respectively, in lines 74-78, 83-88 and 107-110 to add the relevant content of several places.

Comment 2: Line 186-191: the authors found that the 115TH and 116th residues of SEP3 are important for AtSEP3 and AP1 interaction through D-dock and H-dock websites. This prediction is the prerequisite for this paper. However, the detailed prediction process is lacked. Is it predicted by the structure of protein interaction? Could the authors provide the predicted structure of AtSEP3 and AP1 interaction? Please include the website link of D-dock and H-dock websites.

Response: Thank you for your constructive feedback regarding our manuscript. We appreciate the reviewer's request for clarification on the prediction process related to the interaction between AtSEP3 and AP1. In response to this comment, we have made the following revisions to the manuscript: Lines 187-193: We have added detailed information about the model prediction and the protein docking process used to assess the interaction between AtSEP3 and AP1. This includes a brief explanation of the methodology employed for structural prediction and protein docking.Additionally, we have included the relevant links to the D-dock and H-dock websites for further reference.

Comment 3: Line 240-246: why the authors chose the 99th, 101th and 108th residues of SEP3 to mutate as the control? This was not mentioned in the Materials and Methods section nor in the Results section. In addition, Fig. 1, 2 and 4 were also not present in the main text. What does SEP3-3 mean in Fig. 4? I also can't find any data to support the conclusion (AtAP1 and ATAP1 interaction was gradually weakened) at line 242.

Response: Thank you for your valuable feedback and for highlighting several important points regarding our manuscript. We appreciate your thorough review.

In response to your question about the selection of the 99th, 101st, and 108th residues of SEP3 for mutation, we chose these specific residues because leucine zippers play a crucial role in protein interactions. Therefore, we initially mutated all leucines within the K1 domain to investigate their impact on these interactions.

We have ensured that Figures 1, 2, and 4 are now appropriately referenced in the main text to provide clarity. Additionally, "SEP3-3" in Figure 4 refers to a splice variant of the SEP3 gene that lacks the K3 domain.

We also apologize for the error in our original text regarding the interaction between AtAP1 and AtAP1. The correct statement should refer to the interaction between AtSEP3 and AtAP1, and we have revised this in the manuscript to accurately reflect our findings.

Comment 4: Due to the redundancy of *SEP* genes, the abnormal phenotype of single *sep* mutant is subtle. It's better if the authors would try whether the other three SEPs (SEP1, SEP2 and SEP4) could interact with AP1, and if yes, whether the corresponding 116th residue of SEP3 in SEP1, SEP2 and SEP4 is also important?

Response: Thank you for the thoughtful suggestions and insights provided by the reviewer regarding the interactions between SEP1, SEP2, SEP4, and AP1.We would like to inform you that previous studies have already confirmed the interactions between SEP1, SEP2, and SEP4 with AP1. However, due to my recent graduation, further experimental exploration of these interactions, particularly concerning the importance of the corresponding 116th residue in SEP1, SEP2, and SEP4, will be undertaken by my colleague who is continuing this line of research.We sincerely appreciate the reviewer's suggestion, as it provides valuable direction for future investigations.

Comment 5: Besides the AP3/PI/SEP3/AP1 complex, SEP3 also can form complexes with AP3, PI and AG (Theissen et al., 2016; Ruelens et al., 2017). Both complexes are important for regulating petal and stamen development. So whether the 116th residues of SEP3 is also essential for AP3/PI/SEP3/AG complex formation?

Response: Thank you for your insightful question regarding the role of the 116th residue of SEP3 in the formation of the AP3/PI/SEP3/AG complex.

We would like to inform you that our colleagues are currently conducting experiments to investigate this specific aspect. Preliminary results have already been obtained, and we are in the process of analyzing the data further. We will ensure that these findings are included in the revised manuscript once the experiments are completed.

Minor:

Comment 6: Many lines lack the corresponding references, like line 86 "AP1 and AP2 for B-class gene activation", line 191 "confirming earlier findings", lines 257-258 "previous studies show the 120th

leucine”, line 292 “the function of SEP3 and AP1 for floral transition” . Please checkout through the manuscript.

Response: Thank you for your careful review and valuable feedback regarding our manuscript. We appreciate your observation concerning the lack of corresponding references for several statements.

In response to your comments, we have removed the lines that lacked adequate citations, including the references in line 86 (“AP1 and AP2 for B-class gene activation”), line 191 (“confirming earlier findings”), lines 257-258 (“previous studies show the 120th leucine”), and line 292 (“the function of SEP3 and AP1 for floral transition”). Additionally, we have revised and adjusted the introduction section to enhance its clarity and coherence.

We are grateful for your constructive suggestions, which have helped us improve the manuscript.

Comment 7: Line 83: replace ABCDE model with ABCE model, because D-class genes are only confirmed in Arabidopsis and petunia.

Response: Thank you for your valuable feedback regarding our manuscript. We appreciate the reviewer’s suggestion to replace "ABCDE model" with "ABCE model" in Line 83.

We have made the necessary revision to the manuscript, and the term has been updated accordingly to reflect that D-class genes are only confirmed in Arabidopsis and petunia.

Comment 8: I can’t find the Table information (line 189 Table 2.2 and line 191 Table 2.3) in the merged manuscript or any Supplemental data.

Response: Thank you for your careful review of our manuscript and for bringing this issue to our attention. We apologize for the confusion caused by the incorrect table references.

In response to your comment, we have corrected the references from "Table 2.2" and "Table 2.3" to "Supplemental Table 1" and "Supplemental Table 2," respectively. This change ensures that the tables are accurately referenced in the manuscript.

Comment 9: Line 211-212: lack the detailed information about “AtSEP3, AtSEP3-T5 and ATSEP3-DT5” . Line 268: no information was provided for AtSEP3-T5. In addition, all figure legends are too brief to understand the figure for us.

Response: Thank you for your thorough review and helpful comments on our manuscript. We have addressed the concerns raised regarding the lack of detailed information about “*AtSEP3, AtSEP3-T5*, and *AtSEP3-DT5*” in lines 211-212 and the absence of information for *AtSEP3-T5* in line 268.

We have now included the necessary details to clarify these points. Additionally, we have expanded all figure legends to provide more comprehensive explanations, ensuring that each figure is easier to understand.

We greatly appreciate your constructive feedback, which has significantly improved our manuscript.

Comment 10: The sentences in lines 263-267 should be presented in the Material and Methods and the sentences in lines 275- 281 should be presented in the figure legends.

Response: Thank you for your insightful suggestions regarding the organization of our manuscript. We have made the requested revisions to improve clarity and structure.

Specifically, we have moved the sentences from lines 263-267 to the Material and Methods section, and the sentences from lines 275-281 have been incorporated into the figure legends.

We appreciate your guidance in enhancing the quality of our manuscript.

Reviewer #3:

Comment 1: Page 2, At : Shoul be in Italics. Check all the genes reported in the paper

Response: Gene names were changed to italics. Thank you for pointing out the problem, it has been corrected in the appropriate place.

Comment 2: Cite and explain wich are these patways.

Response: Modifications and additions have been made.

Comment 3: MADS transcription factors are generally very similar in amino acid sequences and bioche mical 105properties, yet they possess highly distinct and specific functions.

Response: Modifications and additions have been made.

Comment 4: Reformulate the sentence (not clear).

Response: Modifications and additions have been made.

Comment 5: The flowering process is a critical step for plants transitioning from vegetative growth to [8] ,reproductive growth, with AP1 being a decisive gene for floral bud differentiationtypic ally [9]

.expressed downstream of LFY AP1 is a key gene in flowering induction and plays an i mportant [10] ,role during the transition of the inflorescence meristemand the formation of the flora l meristem is also controlled by this gene.

Response: Modifications and additions have been made.

Comment 6: form DNA-binding dimers and tetramers with many partners, while other MIKC-type MADS domain. transcription factors cannot form floral tetramer-like complexes among themselves. **missing citation.**

Response: Modifications and additions have been made.

Comment 7: At, italic. At, italic. example, add more examples.

Response: revised according to the suggestions of the Reviewer.

Comment 8: The yeast two-hybrid vectors used were pGADT7 and pGBKT7, while overexpression was performed using pCAMBIA1302. For bimolecular fluorescence co mplementation (BiFC), pSPYNE-35S/PUC and pSPYCE-35S/PUC vectors were utilized. The primary strains employed in the experiments included E. coli DH5α, Saccharomyces cerevisiae AH109, and Agrobacterium GV3101. SEP3 sequences were obtained from the NCBI database, and ENDscript was employed for structural prediction to identify the K1, K2, and K3 subdomains with in the K domain. The structure of the AP1 protein was predicted using the AlphaFold 2.1.1 web platfor m. Docking analyses with the existing SEP3 protein structure were conducted on Z-DOCK and HDOCK, revealing that leucine in the K1 subdomain is implicated in the interaction between the two proteins. It is not plant material. Please refer these part to another paragraph.

Response: revised according to the suggestions of the Reviewer.

Comment 9: Add the locus of the two genes (code). At. Add tobacco. Please include the code number (NCBI) of the genes.

Response: revised according to the suggestions of the Reviewer. Thank you very much for your comments, have italicized the section, have added the tobacco growing conditions and gene numbers and trimmed the redundancies. Thank you very much for your comments, the relevant gene numbers have been added as requested. Thank you very much for the pointers, have added the citations

Comment 10: At, At. Restriction mapping of the open reading frames (ORFs) of AtAP1 and AtSEP3 was performed using the MapDraw tool in DNAStar. For pGADT7-AtSEP3, NdeI and EcoRI restriction sites were incorporated into the primers, while for pGBKT7-AtAP1, N deI and BamHI sites were introduced. Similarly, BamHI and SalI sites were added to the primer s for pSPYCE-35S/PUC-AtSEP3, pSPYCE-35S/PUC-Atsep3-T5, and pSPYNE-35S/PUC-AtAP1. Quite common making a cloning. Not necessary.

Response: revised according to the suggestions of the Reviewer.

Comment 11: plasmids were extracted from E.coli strains harboring pSPYCE-35S/PUC-AtSEP3, pSPYCE-35S/PUC-AtSEP3-T5, or pSPYNE-35S/PUC-AtAP1 and introduced into Agrobacterium tumefaciens strain GV3101. This facilitated the subsequent Agrobacterium-mediated transformation of Arabidopsis. Agrobacterium tumefaciens, not E. coli, is used for the transformation of Arabidops is thaliana, so please avoid the use of "facilitate" the agro mediated trasformation. Did you use the floral dip method? If so, please specify this method, or if another approach was used, cite the procedure for generating transgenic A. thaliana plants.

Response: revised according to the suggestions of the Reviewer.

Comment 12: Control and experimental groups were 165prepared with Z-buffer,β-mercaptoethanol, and ONPG. Concentration? Or please refer to a method.

Response: revised according to the suggestions of the Reviewer.

Comment 13: overlapping PCR was employed**. Add the ref. to Table 3**

Response: Thank you for your valuable comments, have attached the relevant configuration method of the solution. It was an oversight in writing at the time that led to the error in this section, and it has been removed. This section has been removed. Thank you very much for your comments regarding the primers for the overlapping PCR in Table1.

Comment 14: Agrobacterium tumefaciens, AS. prepared penetrant. Tobacco. Add to plant material section. How do you growth it? Where? Which condition? Photographed. Please add the model of the microscope and the method used. what is it? be specific. Gentamicin, Ampicillin, and Rifampicin, concentration?, Laser, wavelenght excitation; please indicate how many cells did you use for quantification of each combination. Mutation model prediction. does not make sense. followed by yeast two-hybrid experiments with AtAP1 to identify interaction sites. Additionally, β-galactosidase activity assays and Bimolecular Fluorescence Complementation (BiFC) will be performed to validate the interactions. you alreay have the paragraph of Y2H and BiFC.

Response: Thank you for your comments, the title and content of this section have been changed! Redundancy has been removed. The concentrations have been added, and have put the osmotic solution configurati on method within the article, including the growing conditions of tobacco in the Materials and Methods section. AS is an abbreviation for acetosyringone, the information has been added to the appropriate place

Comment 15: As the previous prediction results and subsequent yeast two-hybridization experiments h ave proved the importance of the 116th leucine in interaction between AtSEP3 and AtAP1. there are results! The section is material and method!!! (Page 8) DH5α strain was used for vector construction. what do you mean? You can make costructs indipendently from the stain.

Response: I apologize for the lack of clarity due to a writing error on my part, and have corrected this portion of the article. Thanks for pointing out the problem, have moved this section to the results section.

Comment 16: (Page 9)the phenotypic characteristics were observed to explore the effects of losing the interaction between AtSEP3 and AtAP1 on the growth and development of Arabidopsis. Wild-type Col-0, sep3 mutant, AtSEP3-101 transgenic plant, overexpressing transgenic line AtSEP3-T5-101in wild-type background and sep3 mutant background were grown in artificial climate chamber. The growth conditions were 16 h light 234/ 8 h darkness, and the growth temperature was 20~25 ^{o}C. please refer to a new pharagraf. It could not be incuded in this one Rif, Gent and Kan, concentration.

Response: Thank you for your comments, the concentration of each reagent has been added. Thank you for your valuable comments, this section has been removed and moved to the appropriate place.

Comment 17: (Page 10)Two restriction sites,SalI and BamH I, were selected according to MCS of the vector, and the flower cDNA of Arabidopsis was used as template in PCR. After digestion, ligation and transformation of E. coli DH5α, the plasmid was extracted and introduced into Agrobacterium tumefaciens GV3101. Finally, the lower epidermis of tobacco leaves was infiltrated and the luminescence was observed, material and method! (Page 10) The purified protein was combined with the probe (TTTTCTAGGGCTTCCATTTTTGGATT TTTTGATTAGCC) at room temperature. material and method.

Response: Thank you for your valuable comments, which have been moved to the Materials a nd Methods section. Thank you for your valuable comments, which have been moved to the Materials a nd Methods section.

Comment 18: (Page 11) Identification of transgenic plants, please indicate how many plants did you grown to do the phenotipic analysis (in material and method section). (Page 11) addition, a little petal-

like structure appeared on the margin of sepals in AtSEP3-T5(sep3) transgenic plants (Fig. 9B). in the image it is not evident.

Response: Modifications and additions have been made.

Comment 19: (Page 11) Discussion. The entire discussion section does not fully engage with interpreting the obtained results to provide insights into what they might imply regarding the flowering mechanism. Currently, this section largely reiterates elements from the introduction rather than focus ing on analyzing and contextualizing the findings within the framework of the flowering p rocess.

Response: Rewriting, modifications, and additions have been made in discussion section. Thank you very much for pointing out the problem, I've made significant changes to the discussion section.

Comment 20: (Page 11): in addition this pharagraph should be "Phenotypical analysis of transgenic plants" or something like that . transgenic plants bolted later: What about the flowring time? Please measure counting the day from germination at which each plant reaches bolting and do the statistics.

Response: Thanks for the advice you provided, have changed the subheading and stated the number of plants to be grown in Materials and Methods. Thank you very much for pointing out the problem, most of the phenotypes are mainly concentrated at the edge of the leaf tip and have been circled in red where there are phenotypes. The statistical methods for flowering time have been updated in the Materials and Methods section and the results are displayed in the line graph in Figure 8B.

Comment 21: (Page 12) In order to prevent premature development of floral organs, SEP3 is inhibited by flowering time proteins AGL24, SVP and SOC1 in addition to the co-inhibitory complex composed ofAP1-SVP [19,20] .AGL24, SEU-LUG and AP1-When SEP3 expression levels finally accumulate through the activation of AP1 and LFY in the third stage of flowers, it begins to synergistically inhibit [21] flowering time genes with AP1. This inhibition may occur through negative feedback regulation of SEP3-SOC1, SEP3-SVP, and SEP3- AGL24 complexes. this part should be mentioned in the introduction.

Response: Thank you for pointing out the problems with the writing of the article, it has been removed and moved to the introductory section.

Comment 22: The flowering process is a key step in the transition from vegetative growth to reproductive growth, and AP1is the decisive gene for flower bud differentiation, It is generally expressed

LFY [23-26] downstream of AP1, a key gene in flowering induction, which plays an important role in the transition of inflorescence meristem, and floral meristem formation is also controlled by this [27-29]. geneAP1 regulates floral meristem formation and floral organ development through temporal and spatial expression, and it promotes early flowering of plants. Introduction.

Response: Thank you for pointing out the problems with the writing of the article, it has been removed and moved to the introductory section.

Comment 23: (Page 15) SEPALLATA1, SEPALLATA2, SEPALLATA3, and SEPALLATA4 (SEP1-4) are 409[37- 40] .essential genes that determine the identity of floral organsTheir overlapping express ion 410patterns control the certainty of floral meristem and floral organ identity. This part is reported in lines 99-103 in the intro.

Response: Thanks for pointing out the writing problem with the article, have removed this section.

Comment 24: Because the luminescence signal could be artificially adjusted, the BiFC assays in Fig. 6 could not illustrate the weakening of interaction between AtSEP3-DT5 and AtAP1. CoIP or in vitro pull-down is better.

Response: Thank you for your valuable feedback on our manuscript. We understand your concerns regarding the BiFC assays. Indeed, due to the potential for the luminescence signal to be artificially adjusted, the BiFC assays have certain limitations in illustrating the weakening interaction between AtSEP3-DT5 and AtAP1.

Due to my upcoming graduation, it is challenging to supplement the relevant experiments (such as CoIP or in vitro pull-down assays) within the available time. However, we have also employed β-galactosidase activity assays in our study to further validate the results of the BiFC experiments. We believe that the combination of these experiments provides support for our conclusions.

I highly value your suggestions and have conveyed them to my colleagues. We plan to continue exploring this topic in future research and will incorporate the relevant experiments to make our findings more robust and credible.

Comment 25: What does "*AtSEP3* transgenic plants" mean in line 297? *SEP3* overexpression?

Response: Thank you for your continued feedback on our manuscript. In response to your question regarding the phrase "AtSEP3 transgenic plants" in line 297, we would like to clarify that it refers to SEP3 overexpression transgenic plants. We have ensured that this is clearly stated in the revised manuscript to avoid any ambiguity.

Comment 26: The experimental design of your study is robust, and the research aims are clearly articulated in the abstract. However, from the Materials and Methods section onward, I observed several gaps in essential details. For example, the tobacco plants used for BiFC assays are not mentioned in the Plant Material section; solution concentrations are missing; protein extraction protocols are not included; and the number of plants analyzed for the phenotypic assessment is not specified. This lack of information makes it challenging to thoroughly evaluate the work.

Response: Modifications and additions have been made.
